# Harnessing Brain Plasticity: The Therapeutic Power of Repetitive Transcranial Magnetic Stimulation (rTMS) and Theta Burst Stimulation (TBS) in Neurotransmitter Modulation, Receptor Dynamics, and Neuroimaging for Neurological Innovations

**DOI:** 10.3390/biomedicines12112506

**Published:** 2024-11-01

**Authors:** Minoo Sharbafshaaer, Giovanni Cirillo, Fabrizio Esposito, Gioacchino Tedeschi, Francesca Trojsi

**Affiliations:** 1 First Division of Neurology, Department of Advanced Medical and Surgical Sciences, University of Campania “Luigi Vanvitelli”, 80138 Naples, Italy; fabrizio.esposito@unicampania.it (F.E.); gioacchino.tedeschi@unicampania.it (G.T.); francesca.trojsi@unicampania.it (F.T.); 2 Division of Human Anatomy, Neuronal Networks Morphology & Systems Biology Lab, Department of Mental and Physical Health and Preventive Medicine, University of Campania “Luigi Vanvitelli, 80138 Naples, Italy; giovanni.cirillo@unicampania.it

**Keywords:** repetitive transcranial magnetic stimulation, theta burst stimulation, neuronal plasticity, neurotransmitters, long-term potentiation, long-term depression, brain-derived neurotrophic factor, receptor, trkB, neuroimaging, neurorehabilitation, neurological disorders

## Abstract

Transcranial magnetic stimulation (TMS) methods have become exciting techniques for altering brain activity and improving synaptic plasticity, earning recognition as valuable non-medicine treatments for a wide range of neurological disorders. Among these methods, repetitive TMS (rTMS) and theta-burst stimulation (TBS) show significant promise in improving outcomes for adults with complex neurological and neurodegenerative conditions, such as Alzheimer’s disease, stroke, Parkinson’s disease, etc. However, optimizing their effects remains a challenge due to variability in how patients respond and a limited understanding of how these techniques interact with crucial neurotransmitter systems. This narrative review explores the mechanisms of rTMS and TBS, which enhance neuroplasticity and functional improvement. We specifically focus on their effects on GABAergic and glutamatergic pathways and how they interact with key receptors like N-Methyl-D-Aspartate (NMDA) and AMPA receptors, which play essential roles in processes like long-term potentiation (LTP) and long-term depression (LTD). Additionally, we investigate how rTMS and TBS impact neuroplasticity and functional connectivity, particularly concerning brain-derived neurotrophic factor (BDNF) and tropomyosin-related kinase receptor type B (TrkB). Here, we highlight the significant potential of this research to expand our understanding of neuroplasticity and better treatment outcomes for patients. Through clarifying the neurobiology mechanisms behind rTMS and TBS with neuroimaging findings, we aim to develop more effective, personalized treatment plans that effectively address the challenges posed by neurological disorders and ultimately enhance the quality of neurorehabilitation services and provide future directions for patients’ care.

## 1. Introduction

Neurological disorders comprise a broad range of diseases that affect the brain, spinal cord, and nerves, leading to debilitating physical, cognitive, and emotional impairments. Conditions such as Alzheimer’s disease (AD), Mild Cognitive Impairment (MCI), Parkinson’s disease (PD), and stroke. millions of people worldwide, often causing chronic and progressive symptoms. Alzheimer’s disease, the top cause of dementia, affects nearly 55 million people globally. In Europe, about 6.9 million individuals have Alzheimer’s, and 15 million have mild cognitive impairment (MCI) ages 50 and older [1]. PD affects nearly 10 million people worldwide, with a prevalence rate of about 1.51 cases per 1000, rising to 9.34 cases per 1000 in those over 60 [2]. Stroke affects 1.12 million people in the European Union each year, and many survivors face long-term disabilities, such as paralysis and cognitive deficits. By 2047, the number of neurological disease incidents in the EU is projected to increase, which highlights the ongoing challenges faced by patients [3].

Despite advances in medicine, many neurological conditions remain poorly managed by pharmacological treatments alone. For example, in Alzheimer’s disease (AD) and mild cognitive impairment (MCI), approximately 6.9 million Americans aged 65 and older are currently affected, with this number projected to rise to 13.8 million by 2060, highlighting the growing impact of these conditions in the absence of preventive or curative treatments [4]. Up to 50% of patients with chronic pain conditions are resistant to standard therapies. Similarly, patients with post-stroke motor impairment often fail to respond fully to drug-based treatments [3]. These limitations highlight the need for novel approaches that can complement or enhance existing treatments. Non-invasive neuromodulation techniques like transcranial magnetic stimulation offer promising changes by targeting specific areas of the brain to promote recovery and neuroplasticity.

Transcranial magnetic stimulation (TMS) is a non-invasive, safe, and painless technique used to activate or modulate cortical targets within the central nervous system (CNS) [5]. TMS coils with electrical currents targeted in specific brain tissue can modulate neuronal activity in a controlled manner, making TMS a powerful tool for diagnostic and therapeutic purposes in neurophysiology [1].

Among the various forms of TMS, repetitive TMS (rTMS) and theta burst stimulation (TBS) have gained significant attention due to their potential for inducing long-term synaptic plasticity. In detail, rTMS involves delivering a series of magnetic pulses over a defined period, and TBS is designed to induce similar effects but in a much shorter duration. TBS is particularly promising because it can achieve the upregulation or downregulation of cortical activity in a fraction of the time required by traditional rTMS, with both techniques potentially improving treatment efficiency [6,7] (Table 1).

In recent years, the application of rTMS and TBS in treating neurological disorders has received increasing attention, and these techniques have shown considerable promise [8]. rTMS influences cortical excitability [9] to modulate neural circuits that are disrupted in these conditions, leading to improvements in clinical symptoms. Furthermore, TBS enhances cortical excitability and cortical inhibition and promotes recovery in conditions characterized by dysfunctional brain networks [10]. rTMS and TBS have demonstrated effectiveness in clinical practice across a range of neurological conditions for brain plasticity and neurorehabilitation [11].

**Table 1:** Key Differences between rTMS and TBS.

Technique	Stimulation Pattern	Effect on Cortical Excitability	Therapeutic Potential
**rTMS**	Repetitive pulses of magnetic stimulation over a specific brain region	Low-frequency: Inhibitory High-frequency: Excitatory	Modulates cortical networks in particular brain regions
**TBS**	Burst of high-frequency magnetic pulses mimicking theta rhythms	Intermittent (iTBS): Excitatory Continuous (cTBS): Inhibitory	Targets neuroplasticity with patterned stimulation

Abbreviation: rTMS: repetitive transcranial magnetic stimulation; TBS: theta burst stimulation; iTBS: intermittent theta burst stimulation; cTBS: continuous theta burst stimulation.

Brain plasticity enables adaptive changes in response to neuromodulation by alteration of synaptic strength, dendritic remodeling, and changes in the excitability of neuronal circuits. Neuroplasticity forms the foundation for cognitive abilities recovery [12]. In recent years, rTMS/TBS have been developed to connect this plasticity for therapeutic purposes. These techniques directly stimulate neuronal populations in targeted brain areas, inducing changes in synaptic efficacy and network dynamics.

Both rTMS and TBS techniques are known to modulate activity and influence the release of neurotransmitter systems and intracellular signaling pathways, mainly gamma-aminobutyric acid (GABA) and glutamate, which play critical roles in inhibitory and excitatory synaptic transmission, respectively. The literature shows that rTMS and TBS alter the function of their associated receptors, such as NMDA (N-methyl-D-aspartate) and AMPA (α-amino-3-hydroxy-5-methyl-4-isoxazolepropionic acid) receptors [13,14,15] (Table 2). Moreover, rTMS/TBS influences the expression of brain-derived neurotrophic factor (BDNF), a key regulator of synaptic plasticity that binds to its receptor, TrkB (tropomyosin receptor kinase B), activating intracellular signaling cascades that promote the growth and differentiation of neurons and synapses [16,17]. This pathway is critical for LTP, which underlies cognitive functions. In addition to BDNF, rTMS and TBS also modulate the expression of various proteins involved in synaptic structure and function, such as presynaptic and postsynaptic density protein 95 (PSD-95), which are essential for the maintenance of synaptic connections [18] (Table 3).

**Table 2:** Effects of rTMS and TBS on Neurotransmitter Systems.

Neurotransmitter System	Role	Effect of rTMS/TBS
GABA (Gamma-Aminobutyric Acid)	Inhibitory synaptic transmission	Modulates GABAergic transmission to decrease cortical inhibition
Glutamate	Excitatory synaptic transmission	Enhances glutamatergic signaling, promoting cortical excitation
NMDA and AMPA Receptors	Synaptic plasticity and transmission	Modulates function of NMDA and AMPA receptors, influencing long-term potentiation (LTP) and long-term depression (LTD)

Abbreviation: GABA: gamma-aminobutyric acid; Glutamate: excitatory synaptic transmission; NMDA and AMPA receptors: N-methyl-D-aspartate and α-amino-3-hydroxy-5-methyl-4-isoxazolepropionic acid receptors.

**Table 3:** rTMS and TBS Modulation of Key Proteins in Synaptic Plasticity.

Protein	Role in Synaptic Plasticity	Effect of rTMS/TBS
BDNF	Promotes neuronal growth and synaptic plasticity	Increases BDNF expression, promoting neuroplasticity and cognitive recovery
TrkB	Receptor for BDNF activates signaling pathways	Modulates synaptic structure and function via activation of intracellular cascades
PSD-95	Maintains synaptic integrity	rTMS/TBS modulates expression, influencing the stability of synaptic connections

Abbreviation: BDNF: brain-derived neurotrophic factor; TrkB: tropomyosin receptor kinase B; PSD-95: postsynaptic density protein 95.

Using neuroimaging techniques for assessing the effects of rTMS/TBS, such as functional Magnetic Resonance Imaging (fMRI), diffusion Magnetic Resonance Imaging (dMRI), Magnetic Resonance Spectroscopy (MRS), Chemical Shift Selective Imaging (CHESS), Positron Emission Tomography (PET), Electroencephalography (EEG), Magnetoencephalography (MEG), and Functional Near-Infrared Spectroscopy (fNIRS), could indicate the real-time effects of rTMS/TBS on brain activity and connectivity (Table 4). Neuroimaging tailors the real-time monitoring of brain activity and connectivity, providing valuable insights into how rTMS and TBS influence neural circuits and biochemical/metabolic alteration in the brain [19]. By combining neuroimaging with rTMS and TBS, researchers can identify biomarkers of treatment response, optimize stimulation protocols, and modify treatments for individual patients.

**Table 4:** Commonly Used Neuroimaging Techniques for Monitoring rTMS and TBS Effects.

Neuroimaging Techniques	Purpose	Advantages
fMRI (Functional MRI)	Measures brain activity and connectivity	Real-time observation of brain regions affected by rTMS/TBS
dMRI (Diffusion MRI)	Assesses structural brain changes	Identifies alterations in white matter pathways
MRS (Magnetic Resonance Spectroscopy)	Analyzes the chemical composition of brain tissue	Provides information on metabolite concentrations in brain regions
CHESS (Chemical Shift Selective Imaging)	Visualizes specific metabolites using selective imaging techniques	Enhances imaging of targeted compounds within brain structures
PET (Positron Emission Tomography)	Measures metabolic processes and neurotransmitter systems	Visualizes the direct effects of rTMS/TBS on neurotransmitter activity
EEG (Electroencephalography)	Monitors electrical activity in the brain	Tracks immediate cortical excitability changes post-stimulation
MEG (Magnetoencephalography)	Records magnetic fields produced by neuronal activity	Provides high temporal resolution for tracking brain activity
fNIRS (Functional Near-Infrared Spectroscopy)	Measures brain hemodynamics through light absorption	Non-invasive, portable, and can be used in naturalistic settings

Abbreviation: MRI: magnetic resonance imaging.

Although the therapeutic potential of rTMS and TBS is confirmed, several challenges remain. One of the primary issues is the variability in patient response to these treatments. While some individuals show significantly improved symptoms and functional outcomes, others exhibit little to no benefit. This variability has been attributed to several factors, including differences in brain anatomy, disease pathology, and individual genetic nature. For instance, genetic polymorphisms in the BDNF gene have been shown to influence the efficacy of rTMS/TBS, with certain variants associated with reduced neuroplasticity (Table 5). Furthermore, the optimal stimulation parameters for different conditions and patient populations need to be clarified; in this condition, it is challenging to make standardized treatment protocols. These techniques represent a much-needed step toward improving outcomes for patients suffering from these life-altering diseases, offering the potential for more personalized and effective care that goes beyond medication alone.

Our study begins with clarifying rTMS and TBS techniques, mainly focusing on neurobiology changes that facilitate/inhibit brain plasticity and plasticity pathway, as an intrinsic capability for use-dependent change, and as a cornerstone of synapse changes and development. Subsequently, our focus shifts to the nuanced alterations observed in rTMS/TBS-derived plasticity measures within prevalent neurological/neurodegenerative diseases. Moreover, our exploration explains the multitude factors influencing rTMS/TBS plasticity measures. This deeper dive into the biological underpinnings enriches our understanding of the intricate interplay between neural circuits and stimulation techniques.

**Table 5:** Factors Influencing Variability in Response to rTMS/TBS.

Factors	Description
Genetic Variability	Polymorphisms in genes like BDNF can influence neuroplasticity and treatment efficacy
Brain Anatomy	Structural differences in cortical regions affect the outcome of rTMS/TBS
Disease Pathology	Different neurological conditions respond variably to brain stimulation techniques

This narrative review aims to provide a comprehensive overview of the current state of knowledge regarding rTMS and TBS, focusing on their role in modulating brain plasticity. Through detailed consideration of the underlying neurobiology, clinical applications, and potential for future research, we hope to contribute to the ongoing efforts to unlock the full therapeutic potential of these innovative neurological techniques regarding rTMS and TBS.

## 2. Fundamental Concepts of the Transcranial Magnetic Stimulation (TMS) Techniques

Transcranial magnetic stimulation (TMS) is a non-invasive method used to stimulate the human brain. It involves generating a brief, high-intensity magnetic field by passing an electric current through a magnetic coil [8]. This magnetic field can excite or inhibit a specific area of the brain located beneath the coil. While TMS can influence various parts of the brain just below the skull, most studies have focused on the motor cortex, where it can induce a focal muscle twitch known as the motor-evoked potential (MEP) [10]. Researchers have utilized TMS to map brain function and assess the excitability of different brain regions by delivering brief pulses of stimulation. These studies have enabled the mapping of sensory, motor, and cognitive functions with high precision of regional neuronal activity to corroborate biophysical models of direct neuronal excitation [8,19]. Repetitive trains of stimulation (rTMS) can modulate the activity of cortical networks, either activating, inhibiting, or altering their function based on factors such as stimulus frequency, intensity, and the configuration of the induced electric field in the brain [20].

### 2.1. Repetitive Transcranial Magnetic Stimulation (rTMS)

The application of rTMS as a strategy to promote plasticity, increase the activity of the neural population, and develop neuromodulation strategies to restore normal neural excitability levels with cell-specific precision could lay the groundwork for transforming current clinical practice [21]. rTMS pulses effectively modify white matter (WM) networks in the human prefrontal cortex, which has the potential for therapeutic applications and rehabilitation [22].

Low-frequency rTMS (LF rTMS) employs repetitive magnetic pulses at frequencies typically below 5 Hz. This protocol is known to induce cortical inhibition and synaptic depression in the targeted brain regions [23]. LF rTMS is considered to have an inhibitory effect, but at low intensities (less than motor threshold, MT), LF rTMS often fails to have measurable effects on motor excitability. Some findings indicate that variability of response to LF rTMS might be related to the level of motor cortex excitability of the targeted muscle. LF rTMS suppresses MEPs only when the target muscle is at rest. The depression of MEP could be increased if LF rTMS is preceded by a high-frequency subthreshold stimulation as compared to no preconditioning stimulus; this increase in cortical depression lasts for at least 60 min [24].

On the other hand, high-frequency rTMS (HF-rTMS) utilizes stimulation frequencies above 5 Hz. This protocol is designed to promote cortical excitation and synaptic potentiation, leading to increased neuronal firing rates and enhanced synaptic transmission with overlap of cortical maps and system dynamics at single neuron and network levels [9]. HF-rTMS (5–25 Hz) at 120% of the MT is thought to increase cortical excitability as demonstrated by the increase in the MEP amplitude size and corticospinal activity [13]. Overall, the differential effects of LF and HF rTMS on cortical excitability provide clinicians and researchers with versatile tools for modulating brain activity and exploring the therapeutic potential of non-invasive neuromodulation techniques [23].

### 2.2. Theta Burst Stimulation (TBS)

TBS (theta-burst stimulation) is a novel therapeutic approach for a wide range of neurological diseases [25]. TBS-related techniques include intermittent theta-burst stimulation (iTBS) and continuous theta-burst stimulation (cTBS). TBS is delivered in bursts of 3 stimuli at 50 Hz, every 2 s, and can be delivered continuously (cTBS) or intermittently (iTBS), inducing a decrease or increase in cortical excitability, respectively [26]. As its name indicates, cTBS involves the application of continuous TBS pulses for 20 to 40 s (300 to 600 stimuli, respectively), mainly inducing long-term depression (LTD), a long-lasting decrease in the strength of synaptic connections [27]. In iTBS, the TBS pattern is applied in a 10 s intertrain interval, with 2 s of stimulation and 8 s of no stimulation, for a total of 190 s, inducing long-term potentiation (LTP), which is characterized by the strengthening of synaptic connections [7].

iTBS is a rTMS protocol that uses a short stimulation period (190 s) and results in increases in cortical excitability persisting beyond the period of stimulation. iTBS has been shown to enhance antioxidative capacity reported as elevated activity of its enzymatic and non-enzymatic components [28]. Although iTBS may be indicative of an appealing technique for modulating cortical plasticity for clinical or therapeutic applications, recent studies have observed that the effect varies greatly between individuals [29].

TBS has shown significant behavioural effects, intending to influence neurological diseases in humans by enhancing brain plasticity, reducing oxidative stress, and modulating inflammation [30]. Investigations have found iTBS to be effective in various conditions like stroke, Parkinson’s disease, and multiple sclerosis by promoting neuroprotection and improving synaptic function [30,31]. In Alzheimer’s disease (AD), iTBS has been especially beneficial, as it reduces oxidative stress, lowers amyloid-beta and amyloid precursor protein levels, and decreases reactive astrogliosis, a contributor to neuroinflammation. These effects help protect neurons and improve cognitive functions, highlighting iTBS as a potential therapy for slowing AD progression [31].

## 3. Rules of Synaptic Plasticity Induced by rTMS/TBS

Synaptic plasticity from rTMS/TBS is shaped by the interaction of neurotransmitters, timing, network remodeling, and postsynaptic potentials, especially in hippocampal neurons. Glutamate activates NMDA and AMPA receptors, driving EPSPs, while GABA mediates IPSPs, balancing synaptic activity. Timing is key, as the precise stimulation relative to neuronal firing dictates LTP or LTD, following principles like STDP [32]. Network remodeling involves long-term changes in synaptic connections from repeated rTMS/TBS, affecting synaptic strength [33]. The balance between excitatory and inhibitory potentials is crucial, with rTMS/TBS enhancing EPSPs or reducing synaptic strength, impacting neuronal network stability.

### 3.1. Neurotransmitter Involvement in Hippocampal Neurons

rTMS and TBS can strongly induce LTP and enhance synaptic plasticity in hippocampal neurons through the activation of glutamatergic receptors, including N-methyl-D-aspartate receptors (NMDARs) and α-amino-3-hydroxy-5-methyl-4-isoxazolepropionic acid receptors (AMPARs) [27]. This activation, even with short stimulation periods ranging from 20 to 190 s leads to the facilitation of LTP in the CA1 region of the hippocampus [33,34]. rTMS/TBS stimulation interventions produce long-lasting effects on cortical physiology and behavior by representing potential rapid neural plasticity, promoting the formation of new synapses, and reorganizing neural networks that support functional recovery [33,34].

### 3.2. Timing-Dependent Effects

The timing of rTMS/TBS stimulation in relation to ongoing critical neural activity on hippocampal plasticity can be influenced by the precise timing of stimulation, reflecting the principles of spike-timing-dependent plasticity (STDP) [35]. Particularly, the timing and duration of TMS modulation regarding LTP- or LTD-like effects are critical factors in plasticity [32]. Timing importance is described by the Hebbian learning rule “fire in response to reward-predicting stimuli” [36] for increasing synaptic transmission (i.e., pre-driving post). Synapses can therefore be punished for a failed attempt at driving a neuron or for a late arrival of the input—both happen when neuron activity rates are high [35,36]. According to the STDP, the human motor cortex significantly demonstrates that motor learning modulates via TMS-induced plasticity and changes in the intact human central nervous system in the context of behavior such as learning and memory [32]. In spiking networks, eligibility traces arise directly from the need to extract a smooth signal from spike trains with the functional bias of cell connectivity that affects the stimulated neurons directly but also has network-wide effects [37]. Changes in connectivity and information processing within the hippocampal network can contribute to plasticity changes [36,37].

### 3.3. Network Remodeling

Network connectivity model changes after stimulation depend on different activation sites in motor cortex neurons and the degree of reorganization caused during stimulation and parameters [38]. The increase in the firing rate is compensated, affecting synaptic plasticity [38] and lasting changes in network connectivity after stimulation [39]. TMS-induced strong depolarization of the superficial excitatory cells of the canonical microcircuit may lead to the recruitment of excitatory neurons and inhibitory neurons, producing a high-frequency repetitive discharge of the corticospinal axons [40,41]. The role of the inhibitory circuits is crucial to entrain and control the firing of the excitatory networks to produce a high-frequency discharge [41], and TBS-activated intracortical networks affect functional connectivity in the motor system [42]. Particularly, BOLD results from fMRI neuroimaging reflect functional connectivity effects, either direct or compensatory [43]. Synaptic activation needs more than 10 times the energy of axonal activation associated with more BOLD changes [44]. Thus, changes in networks and connectivity in various sites of electrical stimulation play important roles in optimizing synaptic plasticity and neurological therapeutic applications [42,43].

### 3.4. Excitatory and Inhibitory Postsynaptic Potentials of Neuron

Neuronal activity is closely tied to membrane potential changes, as the depolarization of the plasma membrane reaches a threshold that triggers the neuron to fire an action potential [45]. An action potential typically involves depolarization of about 100 mV; in contrast, EPSPs depolarize the membrane potential by only 10 to 20 mV, while Inhibitory Postsynaptic Potentials (IPSPs) hyperpolarize the membrane by 5 to 10 mV [45,46]. The hippocampus exhibits well-documented bidirectional synaptic plasticity [46]. Research has shown that high-frequency stimulation of hippocampal Schaffer collateral axons alters EPSPs, enhancing action potential generation in postsynaptic CA1 pyramidal neurons and reducing GABAergic inhibition with enhanced glutamatergic excitability synaptic inputs efficacy [46] with long-lasting enhancement in signal transmission LTP [47]. This results in larger EPSPs and a lowered action potential threshold [48]. Synaptic input can be effectively modulated by activity-dependent changes in ion channels, which influence EPSP amplification, spike threshold, or resting membrane potential, thereby contributing to synaptic modifications [49]. IPSPs are likely comprised of GABA_B_-mediated cortical inhibition activity blockade [50]. However, high-intensity stimulation can generate field potentials [51] and induce a long-lasting decrease in synaptic strength LTD [47].

## 4. Protein Expression in rTMS/TBS

rTMS and TBS impact protein expression in the central nervous system by influencing gene transcription and inducing epigenetic modifications. These techniques generate electromagnetic that penetrate specific cortical areas, creating focal electric currents that modulate neuronal excitability and synaptic transmission through the activation of transcription factors and immediate early genes.

In the detail of transcriptional changes and neuroplasticity, rTMS/TBS influence epigenetic regulators like Deoxyribonucleic Acid (DNA) methylation and histone modifications, which control the accessibility of genes for protein production [52]. This combination of gene expression and epigenetic modulation leads to the synthesis of proteins crucial for neuronal communication, synaptic plasticity, and network connectivity [53]. These molecular effects explain the potential therapeutic benefits of rTMS and TBS for neurological/neurodegenerative diseases. Understanding these mechanisms provides key insights for optimizing their clinical and experimental use. Protein expressions are facilitated, such as:

### 4.1. Genetic Factors

Genetic variations can influence an individual’s responses to rTMS and TBS modulation. Identifying genetic markers associated with therapeutic response, adverse effects, and individual susceptibility is a key focus in neurological treatments [54]. Genome-wide association studies (GWAS) and candidate gene approaches are powerful tools for investigating the impact of genetic factors on the treatment of complex neurological diseases [54,55]. For instance, GWAS has shown genetic influences on cortical inhibition induced by TMS [55]. These approaches are commonly used to explore the genetic basis of rTMS and TBS responsiveness in terms of neural plasticity [55,56].

### 4.2. Gene Expression Changes

**Figure 1:**
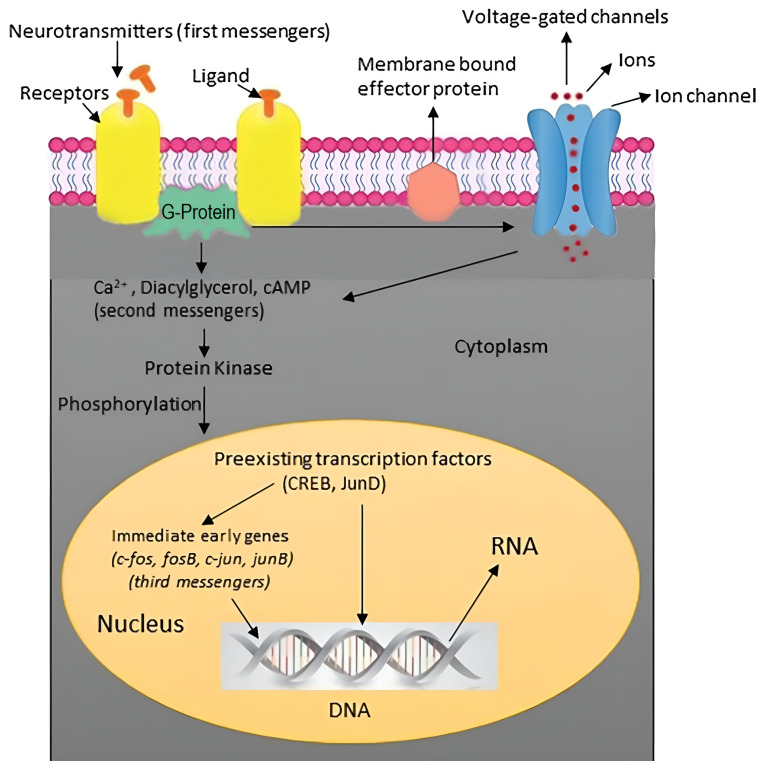
The image depicts a signaling pathway where neurotransmitters bind to membrane receptors, activating G-proteins and effector proteins to produce second messengers like Ca^2^⁺ and cAMP. These messengers activate protein kinases, leading to protein phosphorylation and activation of transcription factors (e.g., CREB, JunD) in the nucleus. This triggers the transcription of immediate early genes, resulting in RNA synthesis and changes in gene expression. Voltage-gated ion channels also play a role in cellular response.

Changes in gene expression within the BDNF-TrkB signaling cascade, particularly in downstream targets related to plasticity, are induced by rTMS/TBS through stimulation of differentiated SH-SY5Y neuroblastoma cells [57]. This leads to the uncoiling of chromatin, making DNA more accessible to transcription factors and thereby increasing gene expression [52] (Figure 1). rTMS and TBS have been shown to induce gene expression changes in the stimulated brain regions. Studies using techniques such as microarray analysis and RNA sequencing have identified alterations in genes related to synaptic plasticity [58], along with an increase in Cholecystokinin (CCK) messenger RiboNucleic Acid (mRNA) expression in several hippocampal and cortical regions, which is functionally relevant to the activation of mesolimbic dopaminergic neuronal pathways [59]. Regarding therapeutic mechanisms of rTMS, a study indicated that HF-rTMS enhances the expression of BDNF by activating the Ca^2+^–CaMKII–CREB pathway in the Neuro-2a cells [60]. Changes in gene expression via BDNF may offer a novel therapeutic approach for treating neurodegenerative and neurological diseases [58,59,60].

### 4.3. Protein Regulation

rTMS/TBS induces prolonged changes in the expression of proteins such as Cyclin-Dependent Kinase 5 (CDK5) and the Postsynaptic Density Protein 95 (PSD-95), which are both involved in modulating synaptic plasticity [52]. Proteomic analyses have revealed alterations in the levels of proteins involved in synaptic function, neuroplasticity, and inflammation following TBS [57]. These protein changes may contribute to the long-lasting effects of neuromodulation on neuronal excitability and connectivity.

### 4.4. Epigenetic Modifications

rTMS and TBS can induce epigenetic changes in the brain, affecting chromatin accessibility and gene expression patterns. These modifications encompass changes in DNA transcription, protein production, and trafficking; alterations in the physical structure of neurons, such as dendritic branches and spines; and ultimately, changes in behavior [14,53]. Studies have shown that using a DNA protein kinase inhibitor significantly enhances precise gene editing, improving integration efficiency and precision by inhibiting DNA polymerase theta. This combined treatment can achieve templated insertions with up to 80% efficiency, with minimal unintended insertions and deletions [61].

## 5. Modulation of Gamma-Aminobutyric Acid (GABA)/Glutamate Neurotransmission and Receptors by rTMS/TBS (Table 6)

Glutamate and GABA are fundamentally important for the development of neuronal circuitry and the maintenance of cognition and behavior [62]. rTMS/TBS may drive changes in excitatory and inhibitory tone through a variety of mechanisms, such as changes to glutamatergic (glutamate transporter and receptor expression) [63] and GABAergic neurons/synapses, neurotrophic factors, or promotion of neurogenesis [64].

Modulation of GABA/Glutamate neurotransmission by rTMS/TBS is crucial for synaptic plasticity; the balance between AMPA and NMDA receptor activity is essential [14], with LTP of AMPA receptor-mediated excitatory postsynaptic potentials (EPSPs) enhanced through NMDA receptor activation. This process adjusts the synaptic response, aligning NMDA receptor-mediated EPSPs with baseline AMPA receptor-mediated EPSPs [15,65]. AMPA receptor-mediated glutamatergic neurotransmission [66]. Notably, a single session of rTMS can produce lasting changes in NMDA receptor and 5-HT1A receptor binding, persisting up to 24 h post-stimulation, which may underlie the therapeutic effects observed in clinical studies involving rTMS and TBS [15]. The synaptic outcome is stimulus-dependent: a single rTMS pulse induces mild NMDA receptor activation, leading to LTD, while a burst of pulses triggers strong NMDA receptor activation, resulting in LTP [67].

### 5.1. GABAergic Mediated Inhibition by rTMS/TBS

Application of LF rTMS to the motor cortex has been shown to affect GABAergic neurotransmission, inducing inhibitory effects by influencing GABAergic interneurons [68] and altering GABA levels in specific brain regions. These effects provide insights into the neurochemical changes induced by this stimulation with greater potential for approaches targeting plasticity or in cases with altered GABAergic responses in neurological dysfunction [69]. The reduced cortical excitability by cTBS has been thought to involve LTD processes and GABAergic mechanisms [24,70].

### 5.2. Glutamate-Induced Excitation by rTMS/TBS

Glutamate is an essential neurotransmitter of activity-dependent synaptic plasticity. HF rTMS applied to certain brain regions is thought to increase cortical excitability [71], and this effect may involve the modulation of glutamate receptors and their release [72].

N-methyl-D-aspartate (NMDA) receptors (NMDARs), which are a type of ionotropic glutamate receptor, play a significant role in synaptic plasticity and are implicated in the mechanisms of rTMS and TBS [15]. Before the stimulation signaling sequence, calcium ions (Ca^2+^) must first become recognizable to other molecules. This happens when Ca^2+^ binds with calmodulin (Ca^2+^); this localization is the main feature of neurogranin, and it could cause enhanced LTP and plasticity [14]. Acutely high Ca^2+^ concentrations activate kinases, while the kinases phosphorylate by modifying the enzymes, regulatory proteins, receptors, and ion channels in the tail of the AMPA receptor, leading to both increased AMPA receptor conductance and insertion into the synapse and LTP [73].

Emerging evidence suggests a dichotomy in NMDAR function that is at least partially explained by the receptor’s subcellular localization; synaptic NMDARs (synNMDARs) activation promotes downstream signaling associated with synapse strengthening and cell survival [74]. NMDARs have been implicated in the mechanisms of TMS, influencing the activity of NMDARs and leading to LTP-like effects [72,75]. Accordingly, the facilitation of cortical excitability produced by iTBS seems to be involved in LTP-like mechanisms related to the modulation of glutamate neurotransmission [6].

#### 5.2.1. rTMS/TBS-Induced Modulation of N-methyl-D-aspartate (NMDA) Receptors

rTMS/TBS induces electrical currents in the targeted brain region, leading to neuronal depolarization that causes the influx of Ca^2+^ through voltage-gated channels, including NMDARs [71,76]. The activation of NMDARs is associated with the release of glutamate; this release contributes to the induction of synaptic plasticity, including LTP-like effects [15], which triggers a series of reactions that lead to LTP and changes in synaptic strength and synaptic plasticity [76,77]. The depolarization and increased neuronal excitability caused by rTMS/TBS involve the activity of sodium (Na^+^), potassium (K^+^), and NMDA ionotropic glutamate receptors. Sodium and potassium ions are fundamental for ion transport across membranes, while NMDA receptors function as ion channels, allowing ions to move through the neuronal membrane [78]. Meanwhile, the influx of calcium through NMDARs is a critical step for the induction of LTP [76,79]. In particular, levels of intracellular Ca^2+^ trigger various signaling cascades within the postsynaptic neuron. Ca^2+^ activates various protein kinases, such as Ca^2+^/calmodulin-dependent protein kinase II (CaMKII) [14], which plays a crucial role in LTP induction [60].

#### 5.2.2. rTMS/TBS-Induced Modulation of α-amino-3-hydroxy-5-methyl-4-isoxazole Propionic Acid (AMPA) Receptors

Alpha-amino-3-hydroxy-5-methyl-4-isoxazole propionic acid (AMPA)/glutamate receptors (AMPARs) are ionotropic glutamates and major excitatory mechanisms underlying various forms of synaptic plasticity [80,81]. The involvement of ions, specifically sodium (Na^+^) and potassium (K^+^), in the context of rTMS and TBS induces electrical currents in the targeted brain region, leading to neuronal depolarization that causes Na^+^ influx through voltage-gated channels, including AMPA receptors [71]. The activation of AMPARs by glutamate, an excitatory neurotransmitter, leads to the influx of Na^+^, resulting in depolarization and the generation of EPSPs and LTP [33]. This process contributes to the overall modulation of neuronal excitability induced by rTMS and TBS. Additionally, rTMS influences K^+^ dynamics across the neuronal membrane, contributing to the overall changes in neuronal excitability [80]. iTBS and cTBS can modulate synaptic plasticity, possibly involving Na^+^ influx and K^+^ dynamics, contributing to the observed changes in synaptic plasticity through AMPARs.

#### 5.2.3. rTMS/TBS-Induced Modulation of Kainate Receptors

While research on rTMS/TBS has predominantly focused on AMPA and NMDA receptors, it is crucial to acknowledge that other glutamate receptors, such as kainate receptors (KARs), can undergo modulation indirectly due to the intricate interactions of synaptic activity [82]. Several kinases have been implicated in the modulation of Ca^2+^ channels with enhanced Ca^2+^ influx due to increased channel open probability [83]. KARs exert significant control over the levels of feed-forward GABAergic transmission onto CA1 pyramidal neurons, thereby increasing the threshold for inducing theta-burst LTP synapse plasticity [84]. On the other side, KARs have presynaptic mechanisms of action, including the regulation of neurotransmitter release, that, once protein Kinase M-ζ facilitates GluA2 recycling in and out of the synapse constitutively, can cause inhibitory synapse plasticity in learning and memory [14,85].

**Table 6:** Modulation of neurotransmitters and receptors by rTMS/TBS.

Study	Cells/Brain Slice/Animal/Human	Methodology (Stimulation)	Targets	Inhibit/Exhibit	Neurotransmitter and Neuroreceptor	Main Results	Interpretation
Ibáñez et al. (2020) [62]	Human	TMS (Single Pulse)	Primary motor cortex (M1)	Inhibit (SICI)	GABA-A receptor	SICI changes depend on the brain state; variations in methodology lead to different outcomes.	Methodology influences the interpretation of SICI results, highlighting sensitivity to brain states.
Moretti et al. (2020) [63]	Animal	rTMS	Striatal regions (related to dopamine release) and glutamatergic systems (cortex-striatal circuits	Exhibit	Glutamate (NMDA), Dopamine	rTMS modulates glutamatergic and dopaminergic pathways, showing potential relevance to substance use disorders.	rTMS may influence pathways involved in addiction, offering potential therapeutic applications.
Moxon-Emre et al. (2021) [64]	Human	rTMS	Dorsolateral Prefrontal Cortex (DLPFC)	Modulate	Glutamate, Glutamine	rTMS changes glutamate/glutamine ratios in young adults with autism.	Supports the potential of rTMS for cortical modulation in autism spectrum disorders.
Kullmann et al. (1996) [65]	Brain Slice (Rat)	Tetanic stimulation or pairing-induced stimulation	Hippocampal CA1 region	Exhibit	Glutamate	Evidence of presynaptic expression and glutamate spill-over at synapses during LTP.	Highlights the role of extrasynaptic glutamate spill-over in synaptic plasticity.
Belardinelli et al. (2021) [66]	Human	TMS (Single Pulse)	Primary motor cortex (hand area)	Modulate	Glutamate	TMS-EEG identified signatures of glutamatergic neurotransmission in the cortex.	Demonstrates how TMS-EEG can reveal glutamatergic activity in human cortical networks.
Huerta and Volpe (2009) [67]	Human	rTMS, TBS	Cortical regions and hippocampus	Modulate	N/A	TMS enhances synaptic plasticity and induces network oscillations.	TMS is shown to enhance brain plasticity, contributing to neural network dynamics.
Dubin et al. (2016) [68]	Human	rTMS	Prefrontal Cortex	Inhibit	GABA	Elevated prefrontal GABA levels post-TMS treatment in patients with depression.	Suggests that TMS-induced GABA increases are associated with treatment efficacy in major depression.
Rafique and Steeves (2020) [69]	Human	rTMS	Visual Cortex	Inhibit	GABA, Glutamate	Low-frequency rTMS affects neurotransmitter concentrations in the visual cortex.	Demonstrates that different rTMS frequencies influence GABA and glutamate differently.
Stoby et al. (2022) [70]	Human	TBS	Visual Cortex	No Effect	GABA, Glutamate	No significant changes in GABA and glutamate concentrations after TBS.	It indicates that TBS does not significantly affect neurotransmitter levels in the visual cortex.
Ciampi de Andrade et al. (2014) [71]	Human	rTMS	Motor cortex (M1) and the dorsolateral prefrontal cortex/premotor cortex (DLPFC/PMC)	Inhibit	Glutamate (NMDA receptor)	rTMS-induced analgesia depends on NMDA receptor activity in pain modulation.	Suggests NMDA receptor involvement in the analgesic effects of rTMS in chronic pain treatment.
Barnes et al. (2020) [72]	Brain Slice (Rat)	TBS	Hippocampus (specifically the CA1 region, stratum radiatum	Exhibit	Glutamate	Relationship between glutamate dynamics and synaptic plasticity.	Highlights glutamate’s key role in synaptic plasticity and learning processes.

**Abbreviations:** TMS, Transcranial Magnetic Stimulation; rTMS, Repetitive Transcranial Magnetic Stimulation; SICI, Short-Interval Cortical Inhibition; TBS, Theta Burst Stimulation.

## 6. Brain-Derived Neurotrophic Factor Gene (BDNF) Neuron Activity and Synaptic Plasticity rTMS/TBS

Neurotrophins are a class of fundamental growth factors expressed within the central nervous system and are identified as fundamental regulators of normal brain development, homeostasis, and plasticity [86]. Brain-Derived Neurotrophic Factor (BDNF) is a widely studied neurotrophin for its linkage to disease and fundamental role in synaptic plasticity [86]. It is believed to be an important regulator of rehabilitation-induced recovery by enhancing synaptic plasticity in neurodegenerative diseases [87]. The interplay between BDNF, neuron activity, and synaptic plasticity is a complex and dynamic process influenced by various factors, including neuromodulation techniques like rTMS and TBS [88].

### 6.1. BDNF Changes

rTMS/TBS stimulation can modulate BDNF levels in the brain and change the developmental expression of glucocorticoid receptors in the brain. It also shows other stress-sensitive behaviors and molecular changes [86] in the brain. Genetic differences have been suggested as a critical contributing factor to neuromodulation-related variability [89]. The BDNF Val^66^Met genetic variation is a relevant factor for inter-individual variability of rTMS/TBS effects on cognitive function and provided novel evidence of the underlying neurophysiological mechanisms subtending such effects [89,90]. High-frequency rTMS has been associated with increased BDNF expression, while low-frequency rTMS may have varying effects [75].

### 6.2. BDNF Alters Neuron Activity Changes and Synaptic Plasticity

BDNF, one of the best-characterized activity-dependent molecules, is expressed in upper-layer neurons and affects cortical wiring by promoting axonal and dendritic growth. Transcriptional regulation of the BDNF gene, which responds to neuronal activity, has also been studied biochemically [91]. rTMS influences neuronal activity by inducing electrical currents in the targeted brain regions. High-frequency rTMS is generally associated with excitatory effects, increasing cortical excitability, while low-frequency rTMS tends to have inhibitory effects [89].

BDNF stands out for its high level of expression in the brain and its potent effects on synapses [16], particularly in terms of synaptic plasticity. The val^66^met polymorphism in the BDNF gene shows less increase in MEP amplitude after motor training than val^66^val carriers. BDNF has been implicated in the control of NMDA receptor-dependent synaptic plasticity and its homeostatic regulation [90]. BDNF regulates synapses, with structural and functional effects ranging from short-term to long-lasting, on excitatory or inhibitory synapses, in many brain regions [16]. The modulation of BDNF and neuron activity by rTMS/TBS can contribute to changes in synaptic plasticity. High-frequency rTMS, by increasing cortical excitability and BDNF levels, may facilitate LTP-like effects, while low-frequency rTMS may induce LTD-like effects [75]. TBS stimulation increases BDNF expression [67]. The BDNF val^66^ met genotype is a major player concerning TBS-induced plasticity and metaplasticity in the human M1_HAND_ [90]_._ Recently, many studies have shown that rTMS and TBS can trigger the expression of brain-derived neurotrophic factors and plasticity-related genes [67].

## 7. Tropomyosin-Related Kinase Receptor Type B (TrkB) rTMS/TBS (Table 7)

The tropomyosin-related kinase receptor type B (TrkB) is a receptor for brain-derived neurotrophic factor (BDNF) and is essential for cellular growth, synaptogenesis, and synaptic plasticity [92]. The TrkB receptor is a member of the family of tyrosine kinase receptors, and when it binds to BDNF, it activates intracellular signaling pathways that play a crucial role in synaptic plasticity, neuronal survival, and overall brain function. rTMS/TBS neuromodulations were effective for TrkB activation; particularly, high-frequency rTMS improves functional recovery, possibly by enhancing neurogenesis and activating the BDNF/TrkB signaling pathway, and conventional 20 Hz rTMS is better than iTBS at enhancing neurogenesis [93].

### 7.1. Neurotrophin-Dependent Plasticity

BDNF, the ligand for TrkB, is one of the neurotrophins that play a key role in synaptic plasticity, learning, and memory [94]. TrkB receptors are of particular importance for a model of more refined mechanisms of memory formation and retention [95]. rTMS/TBS has been shown to modulate the expression and release of BDNF neurotrophins in various brain regions; the TrkB receptor is involved in the downstream signaling cascades initiated by BDNF binding [96]. rTMS/TBS-induced changes in neurotrophin levels may contribute to neuroplastic changes, including synaptic plasticity.

### 7.2. Modulation of Neurotrophins and Effects on Synaptic Plasticity

The regulation of TrkB receptor synthesis is similar to that of BDNF, though to a lesser extent, and is influenced by physiological stimuli like visual input [95]. This is particularly evident in BDNF, where increased neuronal activity boosts expression in neurons with high basal levels and increases the number of neurons expressing BDNF [95,97]. The effects of BDNF are best understood by examining the expression site of the signal-transducing TrkB receptor, whether presynaptic or postsynaptic [97]. Functional changes related to LTP are notable in hippocampal slices, especially in presynaptic structures and functions, following the administration of exogenous BDNF. Research indicates that BDNF-TrkB signaling is crucial for modulating synaptic plasticity, including both LTP and LTD [98]. Techniques like rTMS/TBS can influence BDNF release and TrkB receptor activation, thereby regulating synaptic plasticity in the targeted brain regions [96]. Dysregulation of BDNF-TrkB signaling is associated with various neurological disorders [93]. Consequently, rTMS and iTBS are being investigated for their potential to modulate neurotrophin levels and TrkB activation, significantly promoting neurogenesis in neurological diseases [92].

### 7.3. Hippocampal Plasticity

Hippocampal plasticity is significantly influenced by TrkB, which belongs to the neurotrophin receptor tyrosine kinase family. BDNF is its ligand, and the BDNF/TrkB signaling can modulate LTP/LTD in the hippocampal CA3-CA1 region [99]. Particularly when targeted at the hippocampus, it may influence TrkB-dependent plasticity in the hippocampal region. rTMS/TBS field impact on molecular and cellular processes, including neurotrophin signaling, with effects on synaptic plasticity. TMS neuromodulation participates in regulating structural synaptic plasticity of hippocampal neurons via the activation of BDNF–TrkB signaling pathways in hippocampus [17].

### 7.4. Neurotrophin Signaling and TrkB

TrkB plays a critical role in hippocampal synaptic plasticity by facilitating various intracellular signaling pathways and is essential for neuronal function. The intracellular signaling of activated TrkB is mediated by the recruitment of adaptor proteins that activate well-known signaling cascades like the Ras mitogen-activated protein kinase (Ras/MAPK) pathway and the phosphoinositide 3-kinase pathway, which is important for synaptic plasticity, neuronal survival, and differentiation [99,100]. The human BDNF gene, located on chromosome 11, spans 70 kilobases and has a unique structure with eleven 5-exons and one 3-coding exon, producing 18 mRNA isoforms. In particular, these isoforms, regulated by transcription factors like cAMP-response element binding protein (CREB), enable complex regulation of BDNF expression [99]. The BDNF protein interacts with TrkB receptors, triggering the TrkB signaling cascade, which is crucial for neuronal development, survival, and synaptic plasticity, leading to diverse expression patterns based on stimuli, cell types, and brain regions [94]. BDNF-TrkB signaling via rTMS/TBS was pivotal to the therapeutic effects by leading to LTP and LTD in the DLPFC, prefrontal cortex (PFC), and hippocampus [99,100].

### 7.5. BDNF-TrkB and LTP-LTD Induction

**Figure 2:**
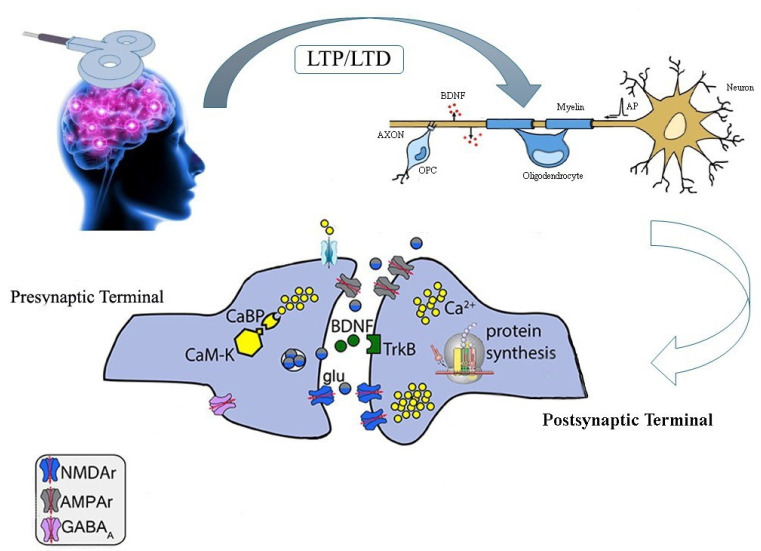
Neural processes in synaptic mechanisms and plasticity, highlighting BDNF’s role. It shows a brain with highlighted neural activity and a brain stimulation device, indicating techniques like LTP and LTD. The diagram includes an axon, myelin sheaths, an oligodendrocyte, presynaptic terminals with proteins, neurotransmitters (e.g., glutamate), receptors (NMDAr, AMPar, GABA_A), and postsynaptic terminals with TrkB receptors binding BDNF, calcium ions, and protein synthesis mechanisms. Arrows indicate neurotransmitter release and signaling directions, emphasizing interactions essential for synaptic function and neural plasticity.

Synaptic plasticity describes the process by which connections between two neurons, or synapses, change in strength. It is a functional term referring to an increase or decrease in synaptic efficacy; synaptic plasticity is thought to be the cellular mechanism for learning and memory. Increasing synapses and connections “LTP” in the hippocampus is the most studied form of synaptic plasticity. BDNF is a small dimeric protein that works through high affinity binding with the receptor tyrosine kinase, TrkB; it is frequently induced by HF-rTMS/iTBS [18,99]. Unexpectedly, a blockade of TrkB receptors (TrkBRs) completely changed the synaptic plasticity induced by chemically induced long-term potentiation (c-LTP), provoking a shift from LTP to LTD in miniature excitatory postsynaptic current (mEPSC) frequency [101]. Generally, BDNF-TrkB signaling has been linked to both LTP and LTD in synaptic plasticity (Figure 2). Notably, rTMS/iTBS upregulates BDNF, synaptophysin (SYN), and postsynaptic density protein-95 (PSD95) expression through activation of the BDNF–TrkB pathway and increases brain 5-hydroxytryptamine, thereby regulating neuroplasticity and improving ejaculation [102].

### 7.6. Synaptic Tagging and Capture Hypothesis

The rTMS/TBS-induced plasticity effects, including those mediated by TrkB, may be related to the synaptic tagging and capture hypothesis. “Synaptic tagging” means synaptic activity generates a tag, which “captures” the PRPs (plasticity-related proteins) derived outside of synapses [103]. This defined that the local ‘tagged’ state and the expression of LTP and LTD are separable processes, especially with major differences in the mechanisms of functional and structural plasticity with specific molecular and structural processes involved in neuron plasticity [104].

**Table 7:** Tropomyosin-related kinase receptor type B (TrkB) brain-derived neurotrophic factor (BDNF) in Neuromodulation.

Study	Methodology (Stimulation)	Targets	LTP/LTD	Neurotransmitter and Neuroreceptor	Main Results	Interpretation
Keifer (2021) [92]	N/A	BDNF (Gene)	N/A	BDNF	Comparative genomics of BDNF gene and its transcriptional regulation across species.	Insight into non-canonical transcription of BDNF and its implications for neurological diseases.
Luo et al. (2017) [93]	rTMS	BDNF/TrkB Signaling, Neurogenesis	LTP	BDNF, TrkB	High-frequency rTMS enhances functional recovery post-ischemia via BDNF/TrkB signaling.	rTMS promotes neurogenesis and recovery through the BDNF-TrkB pathway in ischemic models.
S KS et al. (2024) [94]	N/A	Mitochondria, BDNF-TrkB Signaling	N/A	BDNF, TrkB	BDNF-TrkB signaling modulates mitochondrial function, relevant to neurodegenerative diseases.	Mitochondrial BDNF-TrkB signaling may offer new insights into neurodegenerative disease mechanisms.
Thoenen (2000) [95]	N/A	Neurotrophins, Synaptic Plasticity	LTP	BDNF, NGF	Neurotrophins, including BDNF, are critical for activity-dependent synaptic plasticity.	Highlights the importance of neurotrophins in synaptic plasticity and neural function.
Wang et al. (2011) [96]	rTMS	BDNF-TrkB, Lymphocytes, Brain	LTP	BDNF, TrkB	rTMS enhances BDNF-TrkB signaling in both the brain and lymphocytes.	Provides evidence for peripheral and central effects of rTMS on BDNF-TrkB signaling.
Cohen-Cory et al. (2010) [97]	N/A	Neuronal Connectivity	LTP	BDNF	BDNF is essential for structural neuronal connectivity during development.	Demonstrates the role of BDNF in forming neuronal connections.
Shang et al. (2016) [98]	rTMS	Spatial Cognition, Synaptic Plasticity	LTP	BDNF, Synaptic Proteins	rTMS increases spatial cognition and synaptic plasticity through elevated BDNF and synaptic protein levels.	rTMS boosts cognitive function and plasticity by increasing BDNF in the brain.
Minichiello et al. (2002) [99]	High-frequency electrical stimulation	Hippocampus (CA1 region)	LTP	BDNF, TrkB	TrkB-mediated signaling is necessary for hippocampal LTP.	BDNF-TrkB signaling is critical for long-term potentiation in the hippocampus.
Zhang et al. (2016) [100]	Lipopolysaccharide (LPS)-induced inflammation	Prefrontal cortex, hippocampus, and nucleus accumbens	LTP	BDNF, TrkB	BDNF-TrkB signaling is implicated in inflammation-related depression and may serve as a therapeutic target.	Potential therapeutic relevance of BDNF-TrkB in managing inflammation-linked depression.
Montalbano et al. (2013) [101]	Chemically induced long-term potentiation (c-LTP) via glycine and tetraethylammonium (TEA) chloride.	Hippocampus	LTD	BDNF	Blocking BDNF signaling converts chemically induced LTP into long-term depression (LTD).	Shows how BDNF signaling can reverse synaptic strengthening to weakening (LTD).
Liu et al. (2024) [102]	rTMS	Hippocampus, Sexual Behavior, BDNF-TrkB	LTP	BDNF, TrkB	rTMS via BDNF-TrkB pathway enhances sexual behavior and neuroplasticity in rapid ejaculation rat models.	rTMS improves sexual function and plasticity through BDNF-TrkB signaling.
Lu et al. (2011) [103]	TBS	Hippocampus (CA1 region)	LTP	BDNF, TrkB	TrkB acts as a synaptic and behavioral tag necessary for memory formation and retention.	Identifies TrkB as a key player in memory tagging processes in the brain.
Redondo and Morris (2011) [104]	TBS	Hippocampus (CA1 region), dendrites	LTP	BDNF	Proposes synaptic tagging and capture hypothesis for long-term memory formation.	Provides a theoretical framework for understanding how synapses capture and store memories.
Ma et al. (2013) [17]	rTMS	Hippocampus, Synaptic Plasticity, BDNF-TrkB	LTP	BDNF, TrkB	Magnetic stimulation modulates synaptic plasticity via BDNF-TrkB signaling in cultured hippocampal neurons.	Suggest magnetic stimulation to regulate plasticity through BDNF signaling in neurons.
Lu et al. (2008) [18]	TBS and HFS	Protein Synthesis-Dependent LTP, BDNF	LTP	BDNF	BDNF regulates protein synthesis required for long-term potentiation and memory.	Highlights the role of BDNF in protein synthesis and long-term memory processes.

**Abbreviations:** rTMS, Repetitive Transcranial Magnetic Stimulation; TBS, Theta Burst Stimulation; LTP, Long-Term Potentiation; LTD, Long-Term Depression; BDNF, Brain-Derived Neurotrophic Factor; TrkB, Tropomyosin Receptor Kinase B; NGF, Nerve Growth Factor; HFS, High-Frequency Tetanic Stimulation.

## 8. Ligand G-Protein and Metabotropic Glutamate Receptors mGluR in rTMS/TBS

Ligands, which bind to neurotransmission receptors, typically attach to postsynaptic membrane receptors [105]. G-protein-coupled receptors (GPCRs) are signal proteins that relay signals from cell surface receptors to intracellular effectors and are usually linked with G-proteins. These G-proteins mediate most cellular responses to external stimuli [105], including metabotropic receptors like mGluRs, which respond to glutamate and are specifically located at synaptic and extrasynaptic sites in neurons and glia across nearly all major brain regions [106] As metabotropic GPCRs, mGluRs modulate synaptic transmission and neuronal excitability through intracellular signaling pathways throughout the central nervous system [106,107]. rTMS and TBS primarily induce electric currents in the brain, affecting neuronal excitability and synaptic plasticity. This mechanism differs from traditional ligand-receptor interactions involving specific ligands, G-proteins, or mGluRs, which impact neurotransmission, genetic and protein regulation, and brain network oscillations [67]. A study found that mGluRs are involved in the effects of high-frequency rTMS on NMDAR-dependent glutamatergic transmission in the cortex and independent of slice orientation [108].

## 9. Channel Ion Ionic Imbalance Electrochemical Gradient/Voltage-Dependent Gradient (Resting Level) Channel According to the Gradient rTMS/TBS (Table 8)

### 9.1. Ion Channels

Ion channels, proteins situated within the cell membrane, enable specific ions to traverse the membrane selectively. These channels are essential for upholding the resting membrane potential, initiating action potentials, and aiding in neuronal communication. The function of ion channels, as indicated by an increase in motor threshold and a tendency toward suppression of the recruitment curve, is significant [109]. Cells, including neurons, maintain a specific balance of ions (such as sodium, potassium, chloride, and calcium) inside and outside the cell. This balance is critical for maintaining the resting membrane potential and ensuring proper cellular function [110].

### 9.2. Electrochemical Gradient

The electrochemical gradient is a combination of the electrical gradient (voltage across the membrane) and the chemical gradient (concentration difference of ions across the membrane). It influences the movement of ions across the cell membrane through ion channels. The electrochemical gradient, which includes both the electrical (voltage) and chemical (concentration) gradients, influences the movement of ions across the cell membrane [111], and it has functional roles in active dendritic properties in the processing of synaptic input [73]. rTMS/TBS-induced currents could potentially influence the electrochemical environment and ion movement. Some ion channels are voltage-dependent, meaning their opening and closing are influenced by changes in membrane potential (Figure 1). Voltage-gated sodium channels and voltage-gated potassium channels are crucial for the generation and propagation of action potentials in neurons [83].

### 9.3. Resting Membrane Potential

The resting membrane potential is the voltage difference across the cell membrane when the cell is at rest. Neurons typically have a resting membrane potential due to the unequal distribution of ions across the membrane. Resting membrane potential is crucial for the excitability of neurons. TMS induces magnetic fields that can generate electric currents when applied to the brain. The induced electric currents can influence the excitability of neurons by modulating ion channels and altering membrane potential [112]. TMS may affect voltage-dependent ion channels, including those involved in action potential generation and propagation.

The specific effects of TMS depend on various factors, such as the stimulation parameters (frequency, intensity, duration), the orientation of the magnetic field, and the characteristics of the targeted neural tissue. TMS can influence the activity of neurons by modulating ion channels and altering the electrochemical environment across the cell membrane [110].

### 9.4. Ion Channels and Membrane Potential

Neurons have various types of ion channels, including voltage-gated channels, regulating the flow of ions across the cell membrane, especially in the effect of rTMS/TBS neuromodulation. TMS pulse causes an immediate and transient activation of voltage-gated sodium channels, and the opening and closing of these ion channels contribute to the resting membrane potential and the generation of action potentials [113]. The effects of TBS on ion channels and membrane potential can lead to changes in neuronal excitability. iTBS has been associated with an increase in cortical excitability, possibly mimicking LTP-like effects. cTBS, on the other hand, has been linked to a decrease in cortical excitability, resembling LTD-like effects [110]. TBS-induced effects may involve the modulation of various ion channels, including those responsible for sodium, potassium, calcium, and chloride flux. Voltage-gated calcium channels play a crucial role in synaptic plasticity, and their modulation could influence TBS-induced effects [110]. rTMS/TBS likely influences the activity of ion channels, the resting membrane potential, and the electrochemical gradients in neurons. The specific effects may depend on the TBS protocol (iTBS vs. cTBS) and the unique characteristics of the targeted neural circuits.

**Table 8:** Summary of Findings on Ion Channels, Electrochemical Gradients, and Their Modulation by rTMS/TBS.

Study	Cells/Brain Slice/Animal/Human	Methodology (Stimulation)	Targets	Main Results	Interpretation
Reis J, et al. (2004) [109]	Human	TMS	Motor Cortex Excitability	Levetiracetam modulates ion channels to influence motor cortex excitability.	Levetiracetam influences cortical excitability primarily by ion channel modulation, impacting motor control.
Ye H, et al. (2024) [110]	cells from the buccal ganglia of Aplysia californica	High-frequency magnetic stimulation (micro-magnetic stimulation or µMS)	Cellular Mechanisms	Carry-over effects observed post-stimulation.	Magnetic stimulation induces carry-over effects at a cellular level, which may influence neural excitability.
Hernández-Balaguera E, et al. (2018) [111]	Neuronal membranes (whole-cell patch-clamped cells)	Electrical Circuit Modelling with Whole-Cell Patch-Clamp	GABAergic synapses, Somatostatin (SST) interneurons, Parvalbumin (PV) interneurons, Dentate gyrus (DG), Granule cells (GCs), Medial septum (MS), Protein Kinase A (PKA), Synaptic plasticity	Capacitance distribution identified through fractional-order electrical circuit model.	The study enhances understanding of neuronal membrane capacitance and its influence on excitability.
Pfeiffer F, Benali A. (2020) [112]	Human (brain)	rTMS	Axonal fibers and OPCs	Potential neuroprotective role of NIBS in preventing neuronal degeneration. Ion Channel Re-distribution/Ion Accumulation	Neuromodulation may mitigate the effects of demyelination by preventing harmful ion accumulation and redistribution.
Banerjee J, et al. (2017) [113]	Rat (primary cortical neurons co-cultured with glial cells)	rTMS	Somatosensory cortex, Layer 4/5 pyramidal neurons, Neocortex	Immediate effects were observed in cortical neurons after repetitive stimulation.	Repetitive magnetic stimulation has direct, immediate effects on neuron function, potentially useful for clinical applications.
Su SC, et al. (2012) [83]	Neurons (animal and human studies)	rTMS	N-type Voltage-Gated Calcium Channels and Presynaptic Function	Cyclin-dependent kinase 5 regulates N-type voltage-gated calcium channels affecting presynaptic function.	The study highlights the regulatory role of cyclin-dependent kinase 5 in calcium channel function, with implications for synaptic plasticity.

**Abbreviations:** TMS, Transcranial Magnetic Stimulation; rTMS, Repetitive Transcranial Magnetic Stimulation; OPCs, oligodendrocyte precursor cells.

## 10. Neuroimaging rTMS/TBS (Table 9)

Neuroimaging techniques play a crucial role in advancing our understanding of the effects of rTMS and TBS, especially the combination of neuroimaging with stimulation to characterize the impact of rTMS/TBS on regional brain function, assess the duration of these effects, and determine the functional state of the stimulated region, which might affect the behavioral response on brain structure and function [19,114]. Combining these non-invasive neuromodulation techniques with various neuroimaging modalities allows researchers to investigate changes in neural activity, connectivity, and structure [19], which is increasingly relevant in light of current attempts that seek to increase the efficacy of TMS as a means to induce lasting changes in brain function [114].

### 10.1. Functional Magnetic Resonance Imaging (fMRI)

Functional magnetic resonance imaging (fMRI) can be immensely beneficial in elucidating the neurobiological effects of rTMS and TBS [115], which is widely used to assess changes in regional blood flow and neural activity induced by neuromodulations. Resting-state [116] and task-based activities [117], particularly task-based, are better for understanding the connectome and the effects of rTMS/TBS on connectivity patterns and may contribute to optimizing therapeutic interventions [118]. fMRI can reveal changes in regional excitability and network connectivity/reactivity, beyond the motor cortex, with good spatial resolution [115]. fMRI can provide insights into how rTMS/TBS modulates neural circuits during and after stimulation and their effects in different layers of the brain; there will also be significant insights into the effects on synaptic plasticity [11,119].

#### 10.1.1. Resting-State Functional Connectivity (rsFC)

Functional connectivity and the connectome are two related concepts of neuro and synaptic plasticity and the mapping of nodes within neural networks [120]. TMS can be combined with functional magnetic resonance imaging (fMRI) to investigate resting-state functional connectivity (rsFC) [121]. By applying TMS to a specific brain region, researchers can observe how the stimulation influences the spontaneous activity and connectivity patterns of other brain regions at rest, providing insights into intrinsic network organization. Effective connectivity refers to the causal influence that one neural system exerts over another. TMS can be used to study effective connectivity by applying stimulation to one region and measuring the resulting changes in activity across a network [122]. This helps infer the direction of information flow within the network.

#### 10.1.2. Network Modulation

TMS can be targeted to specific nodes within functional brain networks to investigate and modulate network dynamics. By applying TMS to a particular region, researchers can assess the impact on the connectivity of that region within associated networks, providing information about the network’s organization [123].

#### 10.1.3. Dynamic Causal Modeling (DCM)

DCM is a generic approach for inferring hidden (unobserved) neuronal states from measured brain activity using fMRI, EEG, MEG, and local field potentials (LFPs) [124]. Using dynamic causal modeling (DCM) and Bayesian model selection (BMS) helps find how TMS modulates the directed influences of connectivity within networks of distributed neuronal responses [125].

#### 10.1.4. Mapping and Modulating Functional Hubs

Mapping brain activity and neural connectivity in rodents using optogenetics in conjunction with either functional magnetic resonance imaging (Opto-fMRI) or optical intrinsic signal imaging (Opto-OISI) [126]. By perturbing these hubs with TMS, researchers can study how it affects the overall functional connectivity and communication efficiency within the brain [127]. TMS provides a valuable tool for analyzing and modulating brain connectivity to provide a more comprehensive understanding of the brain’s connectivity patterns.

fMRI enables the mapping of real-time changes in brain activity and functional connectivity, providing insights into how rTMS/TBS modulates neural circuits. It is particularly valuable in identifying regions of the brain where stimulation may yield the most significant therapeutic benefit. By analyzing pre-treatment fMRI data, clinicians can predict patient-specific responses to rTMS/TBS, enabling the development of more individualized treatment protocols. Additionally, task-based and resting-state fMRI allows for the monitoring of changes in network dynamics, thereby helping to optimize stimulation parameters for enhancing therapeutic outcomes.

### 10.2. Magnetic Resonance Spectroscopy (MRS)

Magnetic Resonance Spectroscopy (MRS) offers unique insights into the neurochemical environment of the brain by measuring concentrations of key metabolites, including GABA and glutamate. MRS can reveal imbalances in excitatory and inhibitory neurotransmission that are critical to the mechanisms of rTMS/TBS. MRS can be used to determine other molecules by comparing observed in vivo signals with known ‘fingerprint’ spectra; the frequencies of MRS equipment can be varied to measure resonances from ^1^H (Proton) [128] and provide useful information in planning both biopsy and therapy procedures. rTMS/TBS studies using MRS techniques investigate the impact of neuromodulation on GABAergic concentrations and functional connectivity between the DLPFC and MPFC [68,129]. MRS measures the concentration of various metabolites in the brain, such as neurotransmitters and markers of neuronal activity. It is a method of investigating plasticity in vivo and allows quantification of several neurochemicals, which are identified based on differences in their molecular structure [130]. Pre- and post-treatment MRS data provide a biochemical profile of the brain’s response to stimulation, which can be used to design rTMS/TBS protocols based on the patient’s neurochemical mechanism and baseline. For instance, patients with lower baseline GABA levels may respond differently to stimulation, and MRS could guide clinicians in adjusting frequency, intensity, or target regions accordingly [68,129,130].

In particular, fMRI and MRS offer a powerful, complementary approach to predicting and optimizing patient responses to rTMS/TBS stimulation. Meanwhile, fMRI/MRS not only facilitates making optimal stimulation sites but also enables real-time monitoring and adjustments to maximize neuroplasticity, minimize adverse effects, and improve clinical and therapeutic outcomes. The developing role of fMRI/MRS in this domain highlights the importance of personalized approaches to neuromodulation, where treatment can be precisely applicable to each patient’s unique neural architecture and neurochemical profile.

### 10.3. Diffusion Magnetic Resonance Imaging (dMRI)

Diffusion magnetic resonance imaging (*d*MRI) tractography methods allow for the spatial topography of the white matter, which represents bundles of coherently organized and myelinated axons [131,132]. It might add information on the structural architecture of the brain and be a powerful imaging technique that is a remarkable tool for investigating structural changes in the brain resulting from rTMS/TBS [131,133]. By combining *d*MRI with brain stimulation techniques, researchers aim to elucidate how these interventions impact brain networks and inform the development of targeted plasticity in the brain with a better understanding of therapeutic effects, especially concerning short-term neuroplasticity [133].

### 10.4. Chemical Shift Selective (CHESS)

Chemical shift selective (CHESS) water suppression is a technique used in magnetic resonance imaging (MRI) to suppress signals from fat molecules, allowing for clearer visualization. It suppresses the water signal using frequency-selective 90^o^ pulses [134], followed by a dephasing gradient to minimize the water z-magnetization before excitation [134,135]. Crusher gradients are also applied after each CHESS pulse to null the water transverse magnetization. In the context of rTMS/TBS stimulation, CHESS can be employed to enhance the specificity and accuracy of MRI measurements by reducing artifacts and improving signal-to-noise ratio [136]. It helps better delineate neural structures and discern subtle changes induced by stimulation protocols.

### 10.5. Positron Emission Tomography (PET)

Positron Emission Tomography (PET) imaging is a relevant tool in studying the brain’s physiology. This neuroimaging tool can be used to measure the modulation of specific neurotransmitters and receptor systems, taking advantage of the development of a variety of radioligands for receptors, transporters, enzymatic activity, and neurotransmission processes [27,137]. Various radiotracers can be used to monitor specific proteins, neurochemical processes, and functional connectivity in the living brain non-invasively [137]. PET-rTMS/TBS studies have been heavily focused on evaluating stimulation effects on endogenous dopamine [27,138].

### 10.6. Electroencephalography (EEG)

Electroencephalography (EEG) has permitted experiments designed to non-invasively examine brain states and their dynamics across motor and non-motor cortical areas, including the examination of cortico-cortical interactions on a millisecond time-scale, of normal and abnormal plasticity mechanisms, as well as of interactions between excitatory and inhibitory mechanisms [139]. EEG as the outcome measure enables the implementation of a variety of sophisticated techniques to identify and characterize connectivity networks between different brain regions focusing on neural oscillations and connectivity [12]. It could also facilitate several advances in understanding the pathophysiology and treatment of a variety of neurological conditions [140]

### 10.7. Magnetoencephalography (MEG)

Magnetoencephalography (MEG) can provide rich information for the analysis of dynamic functional brain networks [141] in magnetic fields generated by neural activity following rTMS/TBS. It helps to understand the immediate effects of brain oscillations. MEG can be applied to study the immediate effects of TBS on neural oscillations and assess its impact on network dynamics [142].

### 10.8. Functional Near-Infrared Spectroscopy (fNIRS)

fNIRS is a non-invasive brain imaging technique that uses near-infrared light (650–900 nm) to measure cortical hemoglobin absorptions by detecting deoxygenated (HbR) and oxygenated (HbO) hemoglobin [143]. fNIRS is useful for assessing the effects of rTMS/TBS by measuring neural activity through hemodynamic changes caused by physiological neurovascular coupling on cortical perfusion and hemodynamic responses [143,144]. The integration of fNIRS with rTMS/TBS enhances understanding of the intricate inhibitory or excitatory interplay among distinct cortical regions to optimize treatment parameters and develop targeted interventions for various neurological diseases [145].

**Table 9:** Neuroimaging techniques with rTMS/TBS.

Study	Neuroimaging Type	Stimulation Method	Brain Target	Frequency and Intensity of Stimulation	Main Results	*p*-Value
Kirkovski M, et al. (2023) [115]	Task-based fMRI	TBS	Various cortical areas	High-frequency, repetitive TBS	TBS induces significant neurobiological effects on cortical plasticity and functional connectivity.	*p* < 0.05
Schluter RS, et al. (2018) [116]	Resting-state fMRI	rTMS	Left and right prefrontal cortex	10 Hz, high-frequency rTMS	Differential modulation of resting-state connectivity in left and right prefrontal cortices, with implications for lateralization in neural processing.	*p* < 0.01
Vidal-Piñeiro D, et al. (2014) [117]	Task-based fMRI	Non-invasive Brain Stimulation	Episodic memory network	Variable stimulation frequencies	Task-dependent activity within the episodic memory network predicts memory outcomes, highlighting the role of specific brain circuits in aging populations.	*p* = 0.03
Bhat P, et al. (2023) [118]	Task-based fMRI	rTMS	Supplementary motor area (SMA)	1 Hz, 90% motor threshold	Significant improvement in task-based connectivity in Parkinson’s patients, demonstrating the therapeutic potential of rTMS for motor symptoms.	*p* < 0.05
Sharbafshaaer M, et al. (2023) [11]	Task-based fMRI	rTMS	Dorsolateral prefrontal cortex (DLPFC)	10 Hz, 90% motor threshold	rTMS significantly improves cognitive functions and connectivity within fronto-parietal networks in MCI patients.	*p* < 0.05
Esposito S, et al. (2022) [119]	Resting-state fMRI	rTMS	DLPFC	10 Hz, 90% motor threshold	Enhanced semantic fluency and fronto-parietal connectivity, indicating potential cognitive benefits of rTMS in MCI.	*p* < 0.05
Cohan R, et al. (2023) [120]	Resting-state fMRI	Continuous and intermittent TBS	Primary visual cortex	N/A	Neither continuous nor intermittent TBS modulated resting-state functional connectivity in the visual cortex.	*p* > 0.05
Zhou IY, et al. (2014) [121]	Resting-state fMRI	rTMS	Corpus callosum and interhemispheric cortical areas	N/A	Morphological brain plasticity is foundational to connectivity changes observed in functional neuroimaging studies.	N/A
Lafleur L-P, et al. (2016) [122]	Task-based fMRI	Dual-coil TMS	Various cortical areas	10 Hz, 90% motor threshold	Effective connectivity and plasticity between cortical areas were significantly enhanced, emphasizing rTMS’s role in network reorganization.	*p* = 0.02
Kozyrev V, et al. (2018) [123]	Task-based fMRI	TMS	Visual cortex	High-frequency TMS	TMS induced targeted remodeling of visual cortical maps, demonstrating plasticity within visual processing areas.	*p* < 0.05
Stephan KE, et al. (2010) [124]	Task-based fMRI	Dynamic Causal Modeling (DCM)	Various brain regions	N/A	Provided guidelines for the use of DCM in fMRI to model brain dynamics and causality in connectivity.	N/A
Hodkinson DJ, et al. (2021) [125]	Task-based fMRI	TMS	Motor cortex	High-frequency TMS	Significant plasticity was induced in the operculo-insular and anterior cingulate cortex, as evidenced by changes in task-based connectivity.	*p* = 0.001
Snyder AZ, et al. (2019) [126]	Resting-state fMRI	Structural-Functional Mapping	Various brain regions	N/A	Demonstrated structure-function relationships in the brain, contributing to models of connectivity in resting-state fMRI.	N/A
Jung J, et al. (2020) [127]	Resting-state fMRI	Concurrent TMS/fMRI	Primary motor cortex	N/A	Demonstrated modulation of brain networks through concurrent TMS and resting-state fMRI, focusing on the motor cortex.	*p* < 0.05
Rhodes CJ, (2017) [128]	MRS	MRS Imaging	Atomic nuclei (NMR), unpaired electrons (EPR), and muon particles (μSR)	N/A	MRS provides a detailed examination of brain metabolites, including GABA and glutamate, offering insights into brain function and pathology.	N/A
Cuypers K, Marsman A, (2021) [129]	MRS	Bimodal approach combining TMS with MRS	Primary motor cortex (M1), contralateral motor cortex	TMS paired with MRS to assess motor-cortical plasticity	The bimodal approach allows for a more comprehensive understanding of TMS effects on neurotransmitter systems (GABA, glutamate) and brain plasticity.	N/A
Stagg CJ, (2014) [130]	MRS	TMS, tDCS	Primary motor cortex (M1), primary visual cortex (V1), sensorimotor cortex, and dorsolateral prefrontal cortex (DLPFC)	N/A	MRS reveals changes in GABA levels, contributing to motor-cortical plasticity and offering a tool for studying TMS-induced effects.	N/A
Dubin MJ, et al. (2016) [68]	MRS	TMS	Prefrontal cortex	High-frequency TMS	Elevated prefrontal GABA levels were observed in patients with major depressive disorder (MDD) after TMS treatment.	*p* < 0.05
Amico E, et al. (2017) [131]	dMRI	TMS	Left Precuneus and Left Premotor Cortex	N/A	TMS/EEG-dMRI reveals dynamic interactions between structural and functional connectivity, highlighting brain network plasticity.	N/A
Song SK, et al. (2002) [132]	dMRI	Diffusion MRI	White matter	N/A	Increased radial diffusion without changes in axial diffusion suggests dysmyelination, revealing critical insights into white matter pathology.	N/A
Tavor I, et al. (2020) [133]	dMRI	Diffusion MRI	Left premotor cortex	50 Hz bursts at 80% of the active motor threshold (aMT),	Short-term plasticity in motor areas following motor sequence learning was detected through changes in diffusion MRI metrics.	*p* < 0.05
Haase A, et al. (1985) [134]	CHESS	CHESS Imaging	Not specific	Chemical shift selective	Introduction of CHESS (Chemical Shift Selective) imaging technique in 1H NMR, improving the resolution and specificity of brain metabolite imaging.	N/A
Sanaenezhad F, (2017) [135]	CHESS	MRS Imaging	Not specific	N/A	MRS was proposed as a comprehensive neuroimaging tool for detecting metabolic changes, offering insights into brain health and disorders.	N/A
Baroncini M, et al. (2010) [136]	dMRI and metabolic magnetic resonance imaging	Structural MRI	Hypothalamus	N/A	Showed sex steroid hormone-related structural plasticity in the human hypothalamus, with changes in brain morphology influenced by hormonal factors.	*p* < 0.05
Paus T, et al. (2001) [137]	PET	rTMS to the left mid-dorsolateral frontal cortex (MDL-FC)	Mid-dorsolateral frontal cortex	Repetitive TMS (rTMS), frequency not reported	Demonstrated modulation of cortico-cortical connectivity in the mid-dorsolateral frontal cortex by rTMS.	N/A
Laruelle M, (2000) [138]	PET	Binding competition techniques	Synaptic neurotransmission	N/A	A critical review of in vivo PET techniques for imaging synaptic neurotransmission, emphasizing limitations and insights into receptor competition.	N/A
Aceves-Serrano L, et al. (2022) [27]	PET and MRI	A narrative review of rTMS effects	Various brain regions	Clinical rTMS, frequency/intensity varies	Reviewed PET and MRI findings showing clinical rTMS effects on neurotransmission, functional connectivity, and metabolic changes in the brain.	N/A
Pascual-Leone A et al., 2011 [12]	EEG	Characterization of cortical plasticity and network dynamics	Various cortical regions	Not specified in the summary	Characterized brain cortical plasticity and network dynamics across the age span in health and disease	N/A
Tremblay S et al., 2019 [139]	EEG	Review of clinical utility and prospects	Various cortical regions	Not specified in the summary	Discussed clinical utility and prospects of TMS-EEG	Not specified in the summary
Cash RF et al., 2017 [140]	EEG	Paired-pulse TMS-EEG	Motor and dorsolateral prefrontal cortex	Not specified in the summary	Characterized glutamatergic and GABA(A)-mediated neurotransmission	Not specified in the summary
Liu L, et al. (2022) [141]	MEG	Systematic review of MEG-based dynamic brain network research	N/A	Not applicable (review study)	Provided a comprehensive overview of MEG dynamic brain network studies	Not applicable
Allen CP, et al. (2014) [142]	MRS, MEG	Combined TMS, MRS, and MEG to investigate visual awareness	Visual Cortex	Reversible inhibition of visual cortex	Demonstrated enhanced visual awareness following reversible cortical inhibition	*p* < 0.05
Curtin A, et al. (2019) [143]	fNIRS	A systematic review of integrated fNIRS and TMS research	Not specified	N/A	Reviewed the integration of fNIRS and TMS in studying cortical activation	N/A
Chen SY, et al. (2024) [144]	fNIRS	Meta-analysis of rTMS effects on cortical activity	Cortical regions	Repetitive TMS (parameters not specified)	Demonstrated significant effects of rTMS on cortical activity, evaluated through fNIRS	*p* < 0.01
Hu M, et al. (2021) [145]	fNIRS	Assessment of high-intensity interval exercise effects on brain plasticity	Motor Cortex	High-intensity interval exercise (exact parameters not provided)	Found that short-term high-intensity interval exercise promotes motor cortex plasticity and improves executive function in sedentary females	*p* < 0.05

**Abbreviations:** fMRI, functional magnetic resonance imaging; TBS, Theta-Burst Stimulation; rTMS, Repetitive Transcranial Magnetic Stimulation; MRS, Magnetic Resonance Spectroscopy; dMRI, diffusion magnetic resonance imaging; CHESS, Chemical Shift Selective; PET, Positron Emission Tomography; EEG, Electroencephalography; MEG, Magnetoencephalography; fNIRS, Functional Near Infrared Spectroscopy.

## 11. Neurological Diseases rTMS/TBS (Table 10)

Transcranial Magnetic Stimulation (rTMS/TBS) relies on the stimulation of the motor cortex and the recording of motor-evoked potential in clinical applications [146] for neurological applications, such as Alzheimer’s disease, Mild Cognitive Impairment, Parkinson’s Disease, Multiple Sclerosis, Stroke, Chronic Pain Disorders, Migraine, and Tinnitus. rTMS/TBS protocols are leading to a greater understanding of pathophysiology and the development of novel diagnostic approaches [146,147]. While promising, rTMS/TBS is a new clinical study testing the potential of TMS in various other neurological conditions that appear at a rapid pace [8].

### 11.1. Mild Cognitive Impairment and Alzheimer’s Disease

rTMS/TBS-derived cortical excitability and plasticity measurements serve as diagnostic biomarkers and potential neuroprotective effects of treatment targets for Alzheimer’s disease (AD) and Mild Cognitive Impairment (MCI) [148]. It aims to modulate neural activity and potentially slow down cognitive decline capable of modulating cortical excitability and inducing long-lasting neuroplastic changes. Preliminary findings have suggested that rTMS can enhance performance in several cognitive functions impaired in AD and MCI and modulate brain regions affected by the disease [11,148,149]. rTMS/TBS may be an effective treatment option for patients with MCI [150] and AD, and its potential therapeutic capabilities should be further developed [151].

### 11.2. Parkinson’s Disease

rTMS/TBS has been found to be effective in improving motor dysfunctions and abnormal cortical excitability [152]. Brain activity is believed to underlie motor disturbances in Parkinson’s Disease (PD) [153]. Reversing these abnormalities by rTMS may promote symptom relief and enhance functional recovery by modulating the neural circuits, which may be due to impaired plasticity in the primary motor cortex (M1) in PD [154]. iTBS over the M1 + DLPFC could significantly improve the slowing of gait and has a short-term therapeutic effect on PD [155]. Additionally, rTMS/TBS modulation in the supplementary motor area (SMA) causes excitability that engenders therapeutic effects on motor symptoms in PD to improve motor function [152,154,155]

### 11.3. Multiple Sclerosis

Multiple sclerosis (MS) is an autoimmune disorder of the central nervous system and a major cause of disability, remarkably in young individuals. While non-pharmacological techniques [156], particularly rTMS/TBS, induce long-lasting changes in neuronal circuits to improve clinical status and neurochemical profile in MS [157], rTMS/TBS-like effects provide insights into the neurophysiological aspects of the disease and also produce lower limb spasticity attenuation when neuromodulation is applied using TMS in patients with MS [157,158].

### 11.4. Stroke

Stroke is a leading cause of mortality and disability worldwide, with most survivors reporting motor and cognitive dysfunctions [159]. rTMS/TBS is used in stroke rehabilitation to assess and modulate/modify neural excitability of brain regions [160]. Behind the effect of the LTP neuronal plasticity is the depolarization of pre- and post-synaptic neurons that results in the release of glutamate into the synapse [161]. rTMS/TBS is the main complication, as well as alternative mechanisms related to recovery and mediated stroke rehabilitation [159].

### 11.5. Chronic Pain Disorders

Chronic pain is a debilitating disorder that causes a significant burden to the individual, with effects on their daily life and loss of unpleasant sensory and emotional experiences associated with actual or potential damage [162]. rTMS/TBS has been explored for chronic pain conditions, including neuropathic pain and fibromyalgia. “High-frequency” rTMS (e.g., stimulation frequency ranging from 5 to 20 Hz) to the precentral gyrus (e.g., M1 region) is responsible for attaining a pain relief response through stimulation of enormous distant cortical areas responsible for pain modulation [163]. Stimulation of M1 with elevated frequencies (about 5 Hz) (proof level A) in neuropathic pain shows a definite analgesic impact [164].

### 11.6. Migraine

Migraines cause substantial pain, hypersensitivity to sensory stimuli, nausea, vomiting, and disability. It is one of the most painful conditions experienced by humankind [165]. rTMS/TBS could serve an important adjunctive role in the abortive and preventive treatment of migraine. Stimulation of these peripheral nerves could provide benefits for the treatment of migraine via inhibition [166] of nociceptive transmission in small pain-transmitting fibers and theoretically via modulation of nociceptive activity more centrally in the trigeminal ganglion [165]. It may help reduce the frequency and intensity of migraines by altering normalized excitability brain excitability and neurotransmitter activity in migraine [166,167].

### 11.7. Tinnitus

Tinnitus is described as auditory hallucinations received in the ear without any external stimulation. It affects a patient’s quality of life, is implicated in many problems, and affects the patient’s family [168]. rTMS/TBS aims to modulate auditory pathways to alleviate symptoms by increasing metabolic activation in the auditory cortex in patients, which has shown a highly significant improvement in the tinnitus score and a significant reduction in the tinnitus score [169]. It could be well-tolerated in the majority of patients by hypothetically changing the intrinsic state before brain stimulation. This means there is possibility in the treatment of chronic tinnitus [170].

**Table 10:** rTMS and TBS investigations in neurological diseases.

Reference	Disease	Method	Brian Target	Frequency and Intensity of Stimulation	Main Results	*p*-Value
Sharbafshaaer et al. (2023) [11]	MCI	rTMS	Prefrontal Cortex	Various across studies (e.g., 10 Hz, 20 Hz)	Cognitive functions showed improvement in working memory and executive function	Significant improvement (*p* < 0.05)
Chou et al. (2020) [148]	MCI and AD	rTMS	Dorsolateral Prefrontal Cortex	10 Hz, 110% Motor Threshold	Meta-analysis confirmed modest cognitive enhancement effects	Significant effect size (*p* < 0.01)
Nardone et al. (2014) [149]	MCI and AD	rTMS	Dorsolateral Prefrontal Cortex	20 Hz, 90% Motor Threshold	Demonstrated potential in slowing cognitive decline, particularly in early AD	Not consistently significant (*p* > 0.05)
Cirillo et al. (2023) [150]	MCI	rTMS	Parietal Cortex	10 Hz, 90% Motor Threshold	Long-term improvement in visuospatial abilities and reduced MMP levels	Significant results (*p* < 0.01)
Li et al. (2024) [151]	AD	rTMS	Prefrontal Cortex	10 Hz, 100–120% Motor Threshold	rTMS optimized intervention strategy led to cognitive benefits in AD patients	Significant cognitive improvement (*p*< 0.001)
Hamada et al. (2008) [152]	PD	rTMS	Supplementary Motor Area	High frequency (10–20 Hz), 110% Motor Threshold	Improvement in motor function and reduction of tremors	Significant improvement (*p* < 0.05)
Chung et al. (2020) [153]	PD	rTMS	Motor Cortex	1 Hz, 90–110% Motor Threshold	Enhanced gait performance and balance during gait training	Significant effect (*p* < 0.01)
Bologna et al. (2016) [154]	PD	rTMS	Motor Cortex	1 Hz or higher, variable intensity	Evaluated motor cortex plasticity; relevance in treating motor dysfunction in PD	Mixed results (*p*-values varied)
Cheng et al. (2021) [155]	PD	TBS	Motor and Non-motor Brain Regions	Theta burst stimulation, 80–100% Motor Threshold	Improvement in both motor and non-motor functions, including cognitive and mood	Significant (*p* < 0.001)
Aloizou et al. (2021) [156]	MS	rTMS	Motor Cortex, Prefrontal Cortex	Various frequencies (e.g., 1 Hz, 10 Hz)	Positive effects on spasticity, fatigue, and cognitive functions	Significant for certain outcomes (*p* < 0.05)
Agüera et al. (2020) [157]	MS	rTMS	Prefrontal Cortex	10 Hz, 80–100% Motor Threshold	Study protocol for assessing neurochemical and clinical effects	Study protocol—no results yet
Hulst et al. (2017) [158]	MS	rTMS	Dorsolateral Prefrontal Cortex (DLPFC)	10 Hz, 110% Motor Threshold	Improved working memory performance and increased functional connectivity	Significant (*p* < 0.05)
Sheng et al. (2023) [159]	Stroke	rTMS	Primary Motor Cortex (M1), Trunk Motor Cortex	Variable, theta burst stimulation	Explored neuroinflammatory pathways and recovery mechanisms	Mechanisms explained—no direct *p*-value
Smith and Stinear (2016) [160]	Stroke	TMS	Motor Cortex, Prefrontal Cortex	1–20 Hz, 100–120% Motor Threshold	Evaluated readiness for clinical use, promising for motor recovery	Mixed evidence (*p*-values varied)
Vallejo et al. (2023) [161]	Stroke	rTMS	Motor Cortex, Prefrontal Cortex	Various frequencies, e.g., 1 Hz, 10 Hz	Reviewed rTMS role in neurorehabilitation; highlighted controversies	Mixed results across studies
Aloizou et al. (2021) [156]	MS	rTMS	Motor Cortex, Prefrontal Cortex	Various frequencies (e.g., 1 Hz, 10 Hz)	Positive effects on spasticity, fatigue, and cognitive functions	Significant for certain outcomes (*p* < 0.05)
Barr et al. (2013) [162]	Chronic Pain	rTMS	Motor Cortex, Prefrontal Cortex	Variable frequencies (e.g., 1 Hz, 10 Hz)	GABAergic inhibitory activity measured; potential for chronic pain treatment	Significant inhibition (*p* < 0.05)
Pinot-Monange et al. (2019) [163]	Endometriosis, Chronic Pelvic Pain	rTMS	Motor Cortex	High-frequency (10 Hz), 80–90% Motor Threshold	Reduction in chronic pelvic pain in endometriosis patients	Significant reduction (*p* < 0.05)
Lefaucheur et al. (2014) [164]	Pain and other Neurological Conditions	rTMS	Primary Motor Cortex (M1), Dorsolateral Prefrontal Cortex and Temporoparietal Cortex	1–20 Hz, 80–120% Motor Threshold	Provided therapeutic guidelines for rTMS use in different conditions	Based on clinical trials (varied *p*-values)
Schwedt and Vargas (2015) [165]	Migraine and Cluster Headache	rTMS	Motor Cortex, Occipital Cortex	1 Hz, 100–120% Motor Threshold	Reviewed efficacy of neurostimulation for migraine and cluster headache	Mixed evidence (*p*-values varied)
Brighina et al. (2010) [166]	Migraine with Aura	rTMS	Motor Cortex	High-frequency (10 Hz), 110% Motor Threshold	Restored cortical excitability and reduced migraine frequency	Significant (*p* < 0.01)
Lipton and Pearlman (2010) [167]	Migraine	rTMS	Motor Cortex, Occipital Cortex	Variable frequencies (e.g., 1 Hz, 10 Hz)	Summarized TMS effectiveness in reducing migraine attacks	Significant reduction (*p* < 0.05)
Yang et al. (2023) [168]	Intractable Tinnitus	rTMS	Left Dorsolateral Prefrontal Cortex and Left Temporoparietal Junction	1 Hz, 80–100% Motor Threshold	Brain alterations detected pre- and post-rTMS; improvement in tinnitus symptoms	Significant brain changes (*p* < 0.05)
Kleinjung et al. (2005) [169]	Chronic Tinnitus	rTMS	Auditory Cortex	Low-frequency (1 Hz), 110% Motor Threshold	Long-term reduction in tinnitus symptoms	Significant reduction (*p* < 0.05)
Schoisswohl et al. (2023) [170]	Chronic Tinnitus	cTBS	Auditory Cortex	Theta Burst, 80% Motor Threshold	Feasibility of combining acoustic stimulation and rTMS; reduced tinnitus symptoms	Feasibility proven (no direct *p*-value)

**Abbreviations:** MCI, amnestic mild cognitive impairment; AD, Alzheimer’s disease; PD, Parkinson’s disease; MS, multiple sclerosis; rTMS, repetitive transcranial magnetic stimulation; TBS, theta-burst stimulation; cTBS, continuous theta-burst stimulation.

## 12. Discussion

Neurological and neurodegenerative disorders, such as Alzheimer’s disease, MCI, stroke, Parkinson’s disease, MS, migraine, pain, and Tinnitus, are increasingly prevalent and pose significant challenges to healthcare systems worldwide [1,2,3]. These conditions often have limited effective treatment options, especially when pharmacological interventions alone are insufficient to alleviate symptoms or disease progression. Therefore, exploring novel, non-invasive therapeutic approaches has become a critical goal in neurology and neurorehabilitation. rTMS and TBS are two confirming techniques that offer the potential for targeted brain modulation [6,9]. By directly influencing neural circuits and stimulating brain plasticity, these methods are redefining how neurological conditions can be managed and potentially offering personalized treatment options tailored to individual patient profiles [10,12].

This review highlights the therapeutic potential of rTMS and TBS as advanced tools for fostering brain plasticity, modulating neurotransmitter systems, and enhancing neurorehabilitation outcomes in the most effective neurological and neurodegenerative diseases. These techniques provide promising, non-invasive means of addressing neurological and neurodegenerative disorders by targeting synaptic plasticity and cortical excitability in specific brain areas [8]. The ultimate goal of this research line is to develop and refine neuromodulation methods that not only complement but, in certain cases, surpass pharmacological treatments in effectiveness, especially for conditions that demonstrate limited response to medication alone. By understanding the neurobiological effects of rTMS and TBS, this study aims to inform more personalized and effective interventions that can be adapted to individual patient needs.

A primary challenge in the clinical use of rTMS and TBS lies in the variability of patient responses, which has limited the standardization of treatment protocols. Numerous factors contribute to this variability, including individual genetic differences, neuroanatomical variations, disease-specific characteristics, and baseline brain plasticity levels [88]. For instance, genetic polymorphisms in neurotrophic factors, such as the BDNF Val66Met variant, have been shown to influence neuroplasticity, potentially altering a patient’s response to neuromodulation treatments [89,90]. Similarly, variations in brain structure, particularly within cortical networks, may affect the distribution and effectiveness of induced magnetic fields, further complicating patient outcomes [21,22]. These challenges underscore the need for an in-depth understanding of the molecular and anatomical underpinnings of rTMS and TBS to tailor interventions more effectively [19].

The potential for neuroplasticity to drive improvements in neurological rehabilitation lies in its capacity to reshape and strengthen neural connections, leading to functional recovery in affected brain areas. Neuroplasticity enables adaptive changes in synaptic strength, governed by mechanisms like long-term potentiation (LTP) and long-term depression (LTD), which are essential for cognitive and behavioral functions [15,33]. By fostering these synaptic changes, neuroplasticity promotes the reorganization of neural networks, which is crucial in conditions where certain brain regions may be underactive or compromised due to disease [17,32]. Enhanced neuroplasticity supports recovery across a range of neurological diseases by adapting brain activity to compensate for lost function, improving motor control, cognition, and overall neurological resilience [34]. This adaptive potential makes neuroplasticity a foundational target for therapies aimed at restoring or enhancing brain function in conditions such as stroke, Alzheimer’s disease, MCI, PD, and other neurodegenerative disorders.

This study builds upon the existing literature by delving into the specific effects of rTMS and TBS on the GABAergic and glutamatergic neurotransmitter systems, as well as their influence on key receptors such as NMDA and AMPA receptors, which are critical for LTP and LTD [43]. The modulation of these receptors can lead to structural and functional changes within neural networks, which are crucial for promoting recovery in neurodegenerative conditions [72,74]. This research further investigates the role of brain-derived neurotrophic factor (BDNF) and its receptor, tropomyosin receptor kinase B (TrkB), in rTMS and TBS-induced neuroplasticity [18]. The BDNF-TrkB pathway is central to the maintenance and enhancement of synaptic efficacy, making it a significant focus for understanding how neuromodulation techniques like rTMS and TBS may support long-term recovery [16]. Increased BDNF expression, stimulated by high-frequency rTMS or iTBS, has been associated with improved synaptic plasticity and cognitive outcomes in animal models, and similar effects are being explored in human clinical studies [75,96].

A significant advantage of rTMS and TBS lies in their non-invasive nature and the relative ease with which they can be adapted to individual patients [25]. Compared to surgical interventions or pharmacological treatments, which may come with substantial side effects and variable effectiveness, rTMS and TBS can be administered in a controlled manner with low risk, making them suitable for feasible neurological conditions [23,114]. This adaptability is particularly valuable in a clinical setting, where patients often present with a complex interplay of symptoms that may not respond uniformly to standard treatments [137,139]. By modulating specific cortical areas like DLPFC, PMC, or MPFC, etc., these techniques provide a means of directly influencing affected brain regions [68,71] without systemic drug interactions or invasive procedures.

However, the application of rTMS and TBS in clinical practice is not without limitations. One of the primary constraints is the variability in outcomes, as previously mentioned, which complicates the establishment of universal treatment protocols [140]. Moreover, while neuroimaging tools such as fMRI, MRS, CHESS, and PET have been instrumental in mapping the effects of rTMS and TBS on brain connectivity and neurotransmitter levels, these technologies are not always accessible or practical in routine clinical settings [129]. The high cost and technical expertise required for neuroimaging limit its availability, particularly in resource-constrained environments [131]. Despite these limitations, neuroimaging remains a critical component of rTMS and TBS research, as it provides essential insights into real-time changes in brain activity, enabling a more personalized approach to treatment [144].

From a broader perspective, this line of research has significant implications for the field of neurorehabilitation and neuromodulation. The growing body of evidence supporting the efficacy of rTMS and TBS underscores the potential for these techniques to transform neurorehabilitation, particularly as complementary therapies for conditions like AD, MCI, PD, stroke, MS, chronic pain, migraine, and tinnitus disorders [148,155,157,163,164,166,168]. By enhancing our understanding of the neural mechanisms that underlie rTMS and TBS, this study contributes to a paradigm shift in how we approach brain plasticity and neuroplasticity, emphasizing the role of targeted neuromodulation over traditional, often generalized treatment approaches [11,150]. This shift has the potential to lead to a more nuanced understanding of brain-behavior relationships and to foster innovations in personalized medicine that prioritize patient-specific neuroanatomy, pathology, and genetics [148].

This study’s findings also have the potential to guide future research in several key areas. First, there is a need to explore genetic and molecular markers like blood samples that may predict a patient’s responsiveness to rTMS and TBS, which could help clinicians identify optimal candidates for these therapies [55,163]. For instance, polymorphisms in the BDNF gene have been associated with differential responses to neuromodulation, suggesting that genetic screening could become a valuable tool for personalizing treatment protocols [58]. Additionally, further investigation into the effects of rTMS and TBS on other neurotransmitter systems, such as the dopaminergic and serotonergic pathways, could broaden our understanding of how these techniques influence cognitive and behavioral regulation, potentially extending their applications to a range of neurological and neurodegenerative diseases [63,64].

Moreover, future studies should focus on optimizing the parameters of rTMS and TBS, such as pulse frequency, intensity, and session duration, to maximize therapeutic outcomes [76,85]. While current protocols are generally based on population-level data, there is growing interest in adaptive stimulation techniques that adjust parameters in real time based on patient response, as monitored through neuroimaging or electrophysiological feedback. Such adaptive approaches hold promise for enhancing treatment efficacy and reducing side effects, ultimately contributing to a more refined and individualized approach to neuromodulation.

In terms of clinical applications, rTMS and TBS offer considerable agreement for conditions characterized by deficits in brain plasticity, such as post-stroke motor impairments, cognitive decline in Alzheimer’s disease, MCI, etc. [151,153]. The ability of these techniques to modulate specific neurotransmitter pathways and receptor dynamics suggests they could play a vital role in facilitating recovery and promoting functional reorganization in the brain [152]. Additionally, as the understanding of rTMS and TBS mechanisms continues to grow, there is potential for these methods to be used preventatively in populations at risk of neurodegenerative diseases, providing an avenue for early intervention that could mitigate or delay disease progression [159].

In summary, this review has illustrated the potential of rTMS and TBS to expand the therapeutic landscape for neurological conditions by enhancing brain plasticity through non-invasive means. By deepening our understanding of these techniques’ molecular and neurobiological effects, this study highlights the importance of personalized approaches to neuromodulation, which account for individual genetic, anatomical, and pathological differences. Although challenges remain in standardizing protocols and ensuring accessibility to neuroimaging for treatment monitoring, the evidence underscores the value of rTMS and TBS as versatile tools with broad clinical applications. As research continues to refine these techniques, future studies will likely focus on integrating genetic, imaging, and molecular data to develop optimized, adaptive protocols that maximize therapeutic outcomes and minimize variability in patient response. In this way, rTMS and TBS may lead the way for more effective, tailored interventions in neurorehabilitation and beyond, contributing to the broader goals of personalized and precision medicine in the field of neurology.

## 13. Conclusions

This review emphasizes the substantial therapeutic potential of rTMS and TBS in enhancing brain plasticity and improving functional outcomes across various neurological and neurodegenerative disorders. This study contributes to optimizing rTMS/TBS protocols for clinical application by describing the neurobiology mechanisms and integrating advanced neuroimaging. Future investigations will likely investigate improved and personalized treatment approaches, where neuroimaging techniques such as fMRI, MRS, etc., along with genetic markers, play important roles in guiding patient-specific interventions. These biomarkers could serve as critical tools for maximizing the therapeutic efficacy of rTMS/TBS, allowing for a more targeted and individualized treatment approach. Further research in this area will be essential to expanding the neurobiology scope, especially in the context of BDNF and TrkB signaling alongside LTP and LTD effects via rTMS and TBS techniques and ensuring their effectiveness for a wider range of patients, ultimately advancing the field of non-invasive brain stimulation.

## Data Availability

Not applicable.
